# Volatile ZrO_2_ Antiferroelectric Tunnel Junctions for Rapid, Energy‐Efficient Physical Reservoir Computing

**DOI:** 10.1002/advs.76692

**Published:** 2026-07-23

**Authors:** Taegyu Kwon, Moonseek Jeong, Su In Hwang, Geun Hyeong Park, Joonyong Kim, Hyojun Choi, Hyeong Seok Choi, Da Hyun Kim, Dong Hee Han, Ju Yong Park, Seungho Baek, Jung Ho Yoon, Dong Hyun Lee, Min Hyuk Park

**Affiliations:** ^1^ Department of Materials Science and Engineering & Inter‐University Semiconductor Research Center College of Engineering Seoul National University Seoul Republic of Korea; ^2^ School of Advanced Materials and Engineering Sungkyunkwan University (SKKU) Seoul Republic of Korea; ^3^ Institute of Engineering Research Seoul National University Seoul Republic of Korea; ^4^ Research Institute of Advanced Materials Seoul National University Seoul Republic of Korea

**Keywords:** antiferroelectric tunnel junction, memristor, reservoir computing, short‐term memory, zirconia

## Abstract

Physical reservoir computing requires nonlinear response, fading memory, and rich transient state diversity, yet conventional nonvolatile memories often rely on explicit reset operations or long relaxation times. ZrO_2_‐based two‐terminal antiferroelectric tunnel junctions (AFTJs) exploit the field‐induced tetragonal‐to‐orthorhombic transition and spontaneous back‐switching of antiferroelectric ZrO_2_. This intrinsic self‐relaxation provides reset‐free fading memory in the sub‐ms regime. An amorphous In‐Ga‐Zn oxide interlayer enlarges the dynamic range, and stoichiometric control identifies the 2:2:1‐ZrO_2_ AFTJ as the optimal composition, delivering an I_on_/I_off_ of ∼890, a peak nonlinearity factor of ∼48.4, and a paired‐pulse facilitation index of 1.79. The enhanced memory margin and nonlinear dynamics support 16 transient current states and yield 90.4% accuracy in Modified National Institute of Standards and Technology classification with a fourfold reduction in spatiotemporal dimensionality. Temporal information processing is further assessed using an experimentally calibrated circuit‐level reservoir model, enabling waveform classification, one‐step‐ahead Hénon‐map prediction (normalized root‐mean‐square error [NRMSE] = 0.01489), and forecasting of a noisy real‐world semiconductor index time series (NRMSE = 0.12263). The fabricated 40 000 µm^2^ AFTJ shows a unit latency of ∼2 µs and energy consumption below 480 pJ per operation. Analytical area scaling projects show that a 100 µm^2^ device could achieve ∼192 ns latency and ∼115 fJ per operation.

## Graphical abstract

An antiferroelectric tunnel junction serves as a reservoir computing node, where field‐induced phase transitions and spontaneous ZrO_2_ relaxation deliver nonlinear fading memory. An In‐Ga‐Zn oxide interlayer enlarges the memory margin and multibit state richness. The device enables area‐efficient digit recognition, waveform classification, and chaotic time‐series prediction at sub‐ms speed with pJ‐level energy consumption through self‐resetting antiferroelectric dynamics.

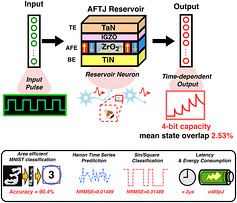

## 1 Introduction

**FIGURE 1:**
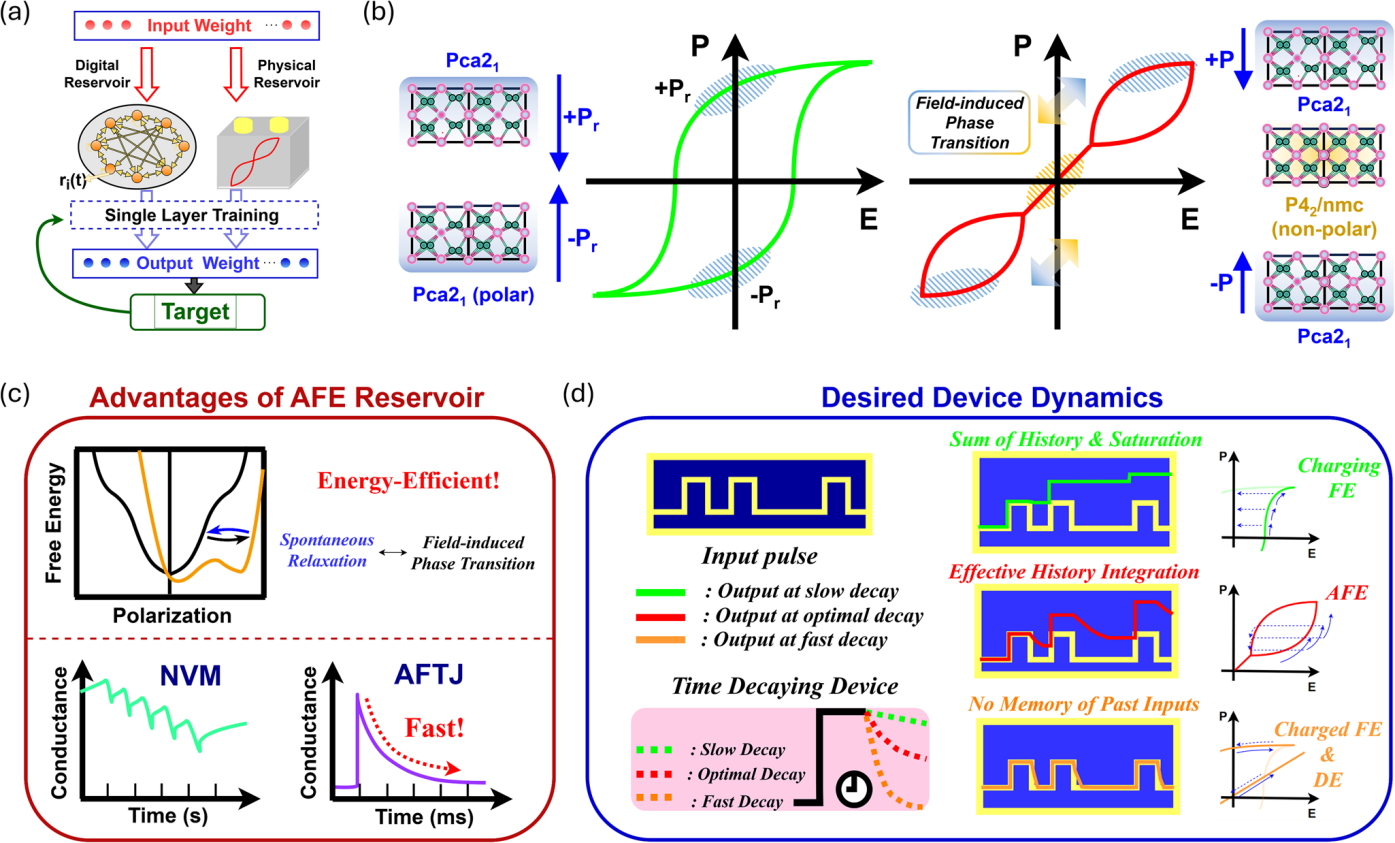
Concept and fundamental principles of antiferroelectric (AFE)‐based physical reservoir computing. (a) Schematic comparison between software‐based digital reservoir computing and the proposed physical reservoir computing, where device dynamics replace recurrent matrix operations. (b) Distinct polarization–electric field (P‐E) responses and corresponding structural phase transitions: (left) conventional nonvolatile polarization switching between polar o‐phases in typical (Hf,Zr)O_2_ ferroelectrics, and (right) the field‐induced reversible phase transition between the non‐polar t‐phase and polar o‐phase characteristic of AFE materials. (c) Key advantages of the AFE reservoir. (d) Desired device dynamics for temporal information processing.

The expansion of edge AI demands low‐latency, energy‐efficient processing of temporal data such as sensory streams and time‐series signals [1]. Recurrent neural networks (RNNs) are widely used for such tasks [2, 3], but training recurrent connections is computationally expensive and energetically inefficient. As an alternative, reservoir computing (RC) has emerged as an efficient computational framework [4, 5, 6]. As illustrated in Figure 1a, RC overcomes the structural limitations of RNNs by completely fixing the internal connection weights [6, 7]. Instead of updating numerous internal weights, the reservoir maps time‐varying inputs into a high‐dimensional nonlinear dynamical space, where temporal correlations are encoded in transient state evolution while only the readout layer is trained. This efficiency makes RC an attractive alternative to RNNs for real‐time temporal data processing.

Despite these algorithmic advantages, software RC implemented on conventional digital hardware still relies on repeated matrix operations and frequent data transfer between separate memory and processing units, remaining limited by the von Neumann bottleneck [8, 9, 10, 11]. Physical reservoir computing (PRC) therefore seeks to exploit the intrinsic dynamics of solid‐state devices so that computation and memory occur simultaneously in the hardware itself (Figure 1a, right panel) [12, 13, 14]. According to the general PRC design framework [15], an effective reservoir device should combine strong nonlinearity [4, 5, 16, 17], short‐term fading memory [18, 19, 20, 21], and rich transient state diversity that expands input signals into distinguishable internal states [16, 17, 22, 23].

A wide range of material‐based reservoir devices has been explored for PRC, including resistive switching memories [24, 25, 26, 27, 28], ferroelectric memristive devices [29, 30, 31, 32], optoelectronic devices [33], and organic memristors [34]. Although these systems can generate high‐dimensional reservoir states, many physical reservoirs rely on current‐mediated state readout or nonvolatile internal states, whose long retention is not always well matched to rapid autonomous temporal processing. A notable exception is the ferroelectric memcapacitive synapse array, which achieved highly energy‐efficient multisensory reservoir operation by using capacitive state modulation with negligible current flux and direct voltage readout [35]. However, this approach represents a distinct capacitive implementation, in which the reservoir dynamics are mediated by capacitively coupled polarization switching and charge‐trapping/detrapping processes rather than by intrinsic volatile structural relaxation. In many current‐read or nonvolatile reservoir devices, sub‐ms operation still requires external reset pulses, which interrupt autonomous dynamics and increase energy consumption.

Here, we introduce scalable and inherently volatile antiferroelectric (AFE) materials based on Zr‐rich HZO and pure ZrO_2_ as a structurally self‐relaxing reservoir platform. Unlike ferroelectric HZO, in which polarization switching occurs between the polar orthorhombic phases (o‐phases, space group *Pca*2_1_) [36, 37, 38], the antiferroelectric response of Zr‐rich HZO or ZrO_2_ originates from an electric‐field‐induced transition between a nonpolar tetragonal phase (t‐phase, space group *P*4_2_/*nmc*) and a polar o‐phase, as shown in Figure 1b. This phase transition and the subsequent spontaneous depolarization enable strong nonlinearity and inherent volatility without requiring intentional reset. Thus, AFE dynamics can provide sub‐ms, reset‐free fading memory for real‐time temporal processing (Figure 1c).

To translate these material‐level advantages into a practical device architecture, we demonstrate physical reservoir neurons based on two‐terminal antiferroelectric tunnel junctions (AFTJs). Specifically, we incorporated an amorphous indium‐gallium‐zinc oxide (a‐IGZO) interlayer to enhance the on/off ratio and expand the available memory margin. By modulating the stoichiometry of the IGZO layer, we optimized the single‐device characteristics to achieve balanced conductance modulation, suppressed leakage current, and stable dynamic operation. To assess its capability for temporal information processing, we performed both classification and prediction tasks using time‐series datasets. These results provide a design strategy for realizing high‐speed and energy‐efficient neuromorphic hardware for real‐time edge computing applications.

## 2 Results

### 2.1 Suitability of Antiferroelectric Dynamics for Rapid PRC

To effectively function as a physical reservoir, a device must possess an optimal short‐term memory (STM) characteristic that appropriately balances state accumulation with relaxation. As conceptually illustrated in Figure 1d, the temporal response of a physical node to sequential input stimuli can be categorized into three distinct regimes: *Simple Sum of History & Saturation*, *Efficient History Integration*, and *No Memory of Past Inputs* [15, 39, 40].

In the *Sum of History & Saturation* regime, the device accumulates the effects of consecutive inputs without meaningful internal decay. This resembles FE charging, where sequential pulses saturate polarization and suppress further dynamic evolution. Once saturated, the device loses its transient state diversity and can no longer encode temporal variations from subsequent inputs, failing to maintain the fading‐memory dynamics essential for continuous RC. Conversely, in the No Memory regime, the state either relaxes too rapidly or fails to encode temporal correlations, akin to a fully charged FE or a linear dielectric (DE) material. The optimal operational window for RC lies in the *Efficient History Integration* regime. Here, the device exhibits a balance between field‐induced state modulation and spontaneous temporal decay, allowing it to retain transient echoes of past inputs while processing new ones without prematurely saturating. We propose that AFE materials intrinsically satisfy this requirement for *Efficient History Integration* due to their field‐induced t‐to‐o phase transition and subsequent spontaneous depolarization. The field‐induced t‐to‐o transition produces stepwise polarization charging, whereas spontaneous o‐to‐t back‐switching provides gradual decay after field removal without instantaneously erasing the device state.

**FIGURE 2:**
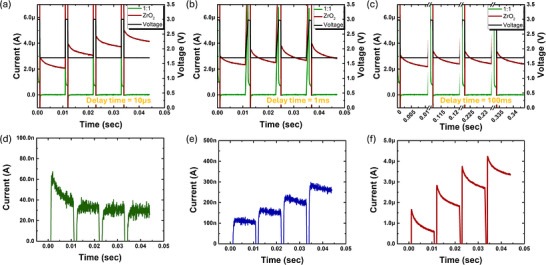
(a–c) Transient current responses of a typical FE Hf_0.5_Zr_0.5_O_2_ (green line) and an AFE pure ZrO_2_ (red line) capacitor under sequential pulse stimulation. A single measurement sequence consists of a 1 ms, 3 V write pulse followed by a 1.7 V read pulse, with zero‐bias delay times of (a) 10 µs, (b) 1 ms, and (c) 100 ms. (d–f) Transient current responses of (d) Hf_0.5_Zr_0.5_O_2_, (e) Hf_0.2_Zr_0.8_O_2_, and (f) pure ZrO_2_ under a four‐pulse train for different (Hf, Zr)O_2_ compositions.

The transient current responses of two distinct material platforms—a FE Hf_0.5_Zr_0.5_O_2_ and an AFE pure ZrO_2_—were investigated. Both materials were fabricated as 6‐nm‐thick TiN/(A)FE/TaN capacitors and subjected to identical sequential pulse stimulation. The measurement scheme consisted of applying a 1 ms, 3 V write pulse, which provides sufficient amplitude and duration to induce full polarization switching in both FE and AFE films. A 1.7 V read pulse was then applied for 10 ms. Crucially, to observe the development of the transient current state, this write‐read pulse pair was repeated four times, with the zero‐bias reset time (delay time) between successive pairs systematically varied across 10 µs, 1 ms, and 100 ms. These time‐dependent current responses are presented in Figure 2a–c, respectively. As observed experimentally, the Hf_0.5_Zr_0.5_O_2_ capacitor exhibits only minimal read current, with negligible history‐dependent variation regardless of the delay time. This indicates that the 1 ms write pulse drives the FE device into a stabilized response regime with negligible history dependence under the present measurement conditions, which we refer to as the *Saturation* or *No Memory of Past Inputs* regime. While such stabilized behavior may be beneficial for storage‐oriented operation, the limited temporal evolution of the response is inadequate for real‐time temporal information processing.

In contrast, the AFE ZrO_2_ capacitor shows pronounced delay‐dependent current modulation, consistent with the behavior required for a dynamic physical reservoir. When the delay time is as short as 10 µs, the AFE capacitor exhibits the highest level of progressive current accumulation across successive pulses. As the delay time increases to 1 ms, this current enhancement begins to attenuate, and at a 100 ms delay, the accumulation effect almost entirely vanishes, indicating that a full spontaneous reset of the AFE state occurs within the 1–100 ms time window. These delay‐dependent current increments indicate that the AFE device possesses dynamic fading‐memory behavior that satisfies the criteria for *Efficient History Integration*. Conventional NVM‐based reservoirs, including typical FE devices, can exhibit efficient history integration when the input interval matches their longer retention times (typically on the order of seconds or longer) as reported in several previous studies [33, 34, 41]. However, such slow relaxation kinetics are inadequate for real‐time edge processing. The AFE device integrates signals and self‐relaxes within the ms timescale. Accordingly, the discussion and evaluation focus on the ms‐to‐sub‐ms regime, where the decay dynamics of AFE materials may offer an advantage over nonvolatile counterparts for rapid temporal signal processing.

To further investigate the correlation between the composition‐dependent phase dynamics and the STM characteristics, we analyzed three different film compositions: Hf_0.5_Zr_0.5_O_2_, Hf_0.2_Zr_0.8_O_2_, and pure ZrO_2_. These 6‐nm‐thick thin films were sandwiched between a top TaN electrode and a bottom TiN electrode. As shown in Figure S1, we measured the current–voltage (*I*–*V*) hysteresis curves of these capacitors (evaluated at 1 kHz with an amplitude of 3 V) in both their pristine states and after 10 000 cycles of electrical pulse repetition (using 3 V, 50 kHz pulses). The results demonstrate that as the composition shifts from a 1:1 ratio toward a more Zr‐rich stoichiometry, the AFE‐like behavior becomes progressively more pronounced, as evidenced by the systematic evolution of the back‐switching peak. In particular, after 10 000 wake‐up cycles, the back‐switching peak in the positive‐voltage region appears near 0 V for Hf_0.2_Zr_0.8_O_2_, whereas it is shifted to around 1.0 V for pure ZrO_2_. This distinction indicates that Hf_0.2_Zr_0.8_O_2_ still retains an MPB‐like character and does not yet exhibit fully developed AFE behavior, while the ZrO_2_ film shows a more complete antiferroelectric response.

Furthermore, the compositional dependence of the crystallographic phase evolution was systematically investigated using grazing incidence X‐ray diffraction (GIXRD) (Figure S2 and Tables S1 and S2). To exclude the contribution of the top electrode and directly probe the structural characteristics of the dielectric layer, the GIXRD analysis was performed on HZO and ZrO_2_ capacitor stacks fabricated without the TaN capping layer. As the Zr atomic fraction increases from Hf_0.5_Zr_0.5_O_2_ to pure ZrO_2_, the diffraction spectra exhibit a clear and monotonic suppression of the monoclinic (m‐) phase. For quantitative analysis, the GIXRD profiles were deconvoluted into three phase‐related components, namely 111_m_, 111_o_/101_t_, and 002_o_/110_t_. Hereafter, hkl_x_ stands for (hkl) diffraction from x‐phase, where x can be o, t, and m. The relative m‐phase fraction was estimated semi‐quantitatively from the fitted peak‐area ratio $A_{111_{m}}/\left(A_{111_{m}}+A_{111_{o}/101_{t}}+A_{002_{o}/110_{t}}\right)$, which decreases from 11% in the Hf_0.5_Zr_0.5_O_2_ film to 3% in the Hf_0.2_Zr_0.8_O_2_ film and reaches 0% in the pure ZrO_2_ sample (Table S1) [42]. This pronounced suppression of the m‐phase is attributed to the reduced crystallization thermal budget in Zr‐rich compositions during rapid thermal annealing without top‐electrode capping, which suppresses the formation of the thermodynamically stable m‐phase and instead favors direct crystallization into the non‐monoclinic t‐ and/or o‐phases.

This compositional phase evolution is further reflected in the lattice aspect ratio, defined as c/a for t‐phase and 2a/(b + c) for o‐phase. In the present analysis, the aspect ratio was extracted from the fitted peak positions of 111_o_/101_t_ and 002_o_/110_t_. Because the t‐phase generally exhibits a smaller aspect ratio than the o‐phase, the aspect ratio can be used as a comparative structural descriptor for tracking the relative balance between the competing t‐ and o‐phases [43, 44]. Our quantitative analysis reveals a systematic decrease in the aspect ratio with increasing Zr content, from 1.014 for Hf_0.5_Zr_0.5_O_2_ to 1.006 for Hf_0.2_Zr_0.8_O_2_ and further to 0.997 for pure ZrO_2_ (Table S1), indicating a progressive reduction in the relative o‐phase contribution and a corresponding increase in t‐phase dominance. Although the absolute aspect ratio of the pure ZrO_2_ film is lower than the theoretical aspect ratio of bulk‐like fluorite oxides (∼1.02–1.03 for t‐phase and ∼1.03–1.04 for o‐phase), similarly reduced values near unity have also been reported in thin‐film HfO_2_‐ZrO_2_ systems, where the extracted lattice metric can be strongly affected by residual stress, thermal expansion mismatch, and the limited separation of overlapping o/t diffraction peaks in ultrathin films [42, 45, 46]. Therefore, in the present system, the absolute value itself should be interpreted with caution, whereas the monotonic compositional trend remains physically meaningful and is consistent with the gradual phase evolution toward a more t‐phase‐dominant structure.

This interpretation is also supported by the evolution of the unit‐cell volume. As the Zr content increases, the extracted unit‐cell volume decreases from 133.02 to 130.85 Å^3^ (Table S2, Note S1), indicating lattice densification [47]. Taken together, the monotonic suppression of the m‐phase, the systematic decrease in aspect ratio, and the continuous unit‐cell densification provide consistent and quantitative evidence that increasing the Zr concentration shifts the structural balance toward a t‐phase‐dominant non‐m‐phase state while progressively reducing the relative contributions of the o‐ and m‐phases.

This observation is consistent with the established thermodynamic picture that Zr‐rich compositions favor the non‐polar t‐phase over the polar o‐phase due to its lower crystallization temperature and compact lattice structure. The impact of this structural t‐phase stabilization on temporal data processing is evident in the transient current responses under four sequential pulse stimulations across the different compositions (Figure 2d–f), where the Zr‐rich devices exhibit the optimal balance between field‐induced polarization and rapid spontaneous relaxation; the detailed pulse scheme and the timing of transient current acquisition are described in Section 4 (Experimental Section). A quantitative comparison of the paired‐pulse facilitation (PPF) indices, defined from the transient current response and calculated as described in Section 4, provides insight into the depolarization kinetics of these materials. For the Hf_0.5_Zr_0.5_O_2_ composition, nonvolatile ferroelectricity causes the PPF index for the initial segment to drop below unity (0.629), followed by a convergence toward one in subsequent segments. This saturation indicates that the FE polarization is significantly switched by the first write pulse; thereafter, the device fails to effectively integrate new temporal signals, acting mainly as a static capacitor undergoing dielectric charging.

In contrast, the Zr‐rich capacitors (Hf_0.2_Zr_0.8_O_2_ and pure ZrO_2_) maintain PPF indices well above unity (ranging from ∼1.24 to 1.36, and from ∼1.27 to 1.70, respectively) throughout subsequent pulse sequences. This sustained facilitation reflects an optimal balance between field‐induced polarization and the rapid spontaneous back‐switching inherent to the AFE state. The fast depolarization of the metastable o‐phase prevents premature saturation, endowing the devices with the dynamic fading memory necessary to integrate consecutive temporal inputs. These results identify AFE t‐to‐o transition and back‐switching as the material basis for STM reservoir operation.

### 2.2 Single‐Device Performance of ZrO_2_‐IGZO Bilayer AFTJs

While Section 2.1 demonstrated the compositional dependence of antiferroelectricity and the resulting STM behavior in HZO films, bare capacitors provide insufficient memory margin for PRC. As revealed in Figure S3, both the Hf_0.2_Zr_0.8_O_2_ and ZrO_2_ capacitors exhibit a limited memory margin, which restricts the state diversity required for a reservoir system. To overcome this limitation and enhance I_on_/I_off_, we engineered the device stack by inserting an a‐IGZO layer on top of the AFE film. This integration yields an AFTJ device with an enlarged memory margin (see Figure S3 for details). Furthermore, because the electrical properties of amorphous IGZO are highly sensitive to its stoichiometry [48, 49, 50], we systematically modulated the IGZO composition to optimize and quantify the electrical characteristics of the individual AFTJ devices, with emphasis on the ZrO_2_ AFTJ because it provides a larger memory margin.

**FIGURE 3:**
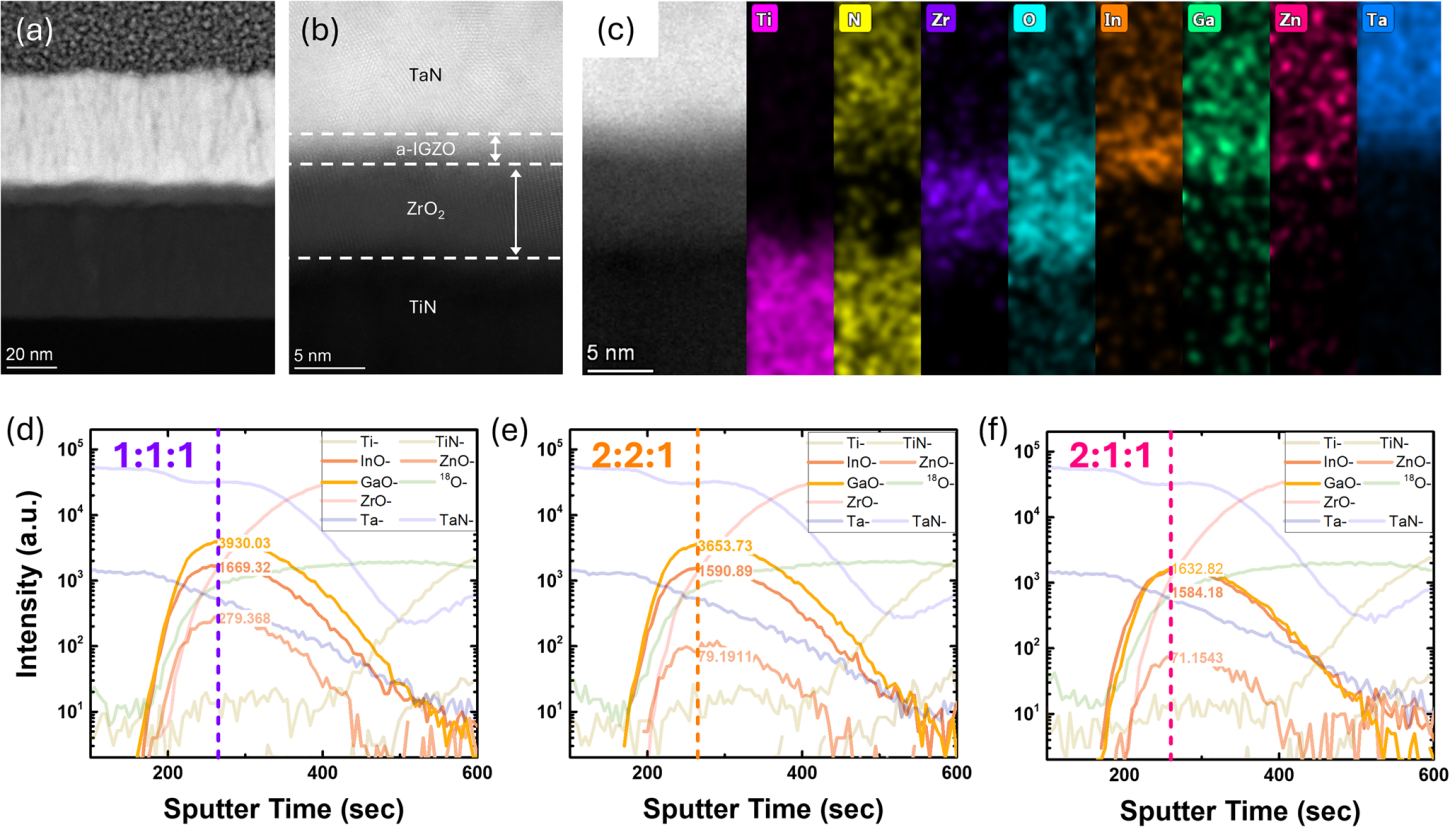
Structural and compositional characterization of the engineered AFTJ stack. (a) Cross‐sectional scanning transmission electron microscopy (STEM) image of the integrated AFTJ device. (b) High‐resolution STEM image confirming the well‐defined heterostructure consisting of a 6‐nm‐thick AFE ZrO_2_ layer and a 1.8‐nm‐thick a‐IGZO interlayer, sandwiched asymmetrically between the bottom TiN and top TaN electrodes. (c) Corresponding energy‐dispersive X‐ray spectroscopy (EDS) elemental mapping of the AFTJ cross‐section. (d–f) Time‐of‐flight secondary ion mass spectrometry (ToF‐SIMS) depth profiles of the AFTJ stacks with varying IGZO stoichiometries. The dashed lines indicate the peak intensity region of the IGZO interlayer. The relative intensity ratios of the InO^−^, GaO^−^, and ZnO^−^ signals systematically reflect the engineered atomic compositions: (d) In:Ga:Zn = 1:1:1, (e) 2:2:1, and (f) 2:1:1.

The engineered AFTJ features a stack comprising a 6‐nm‐thick ZrO_2_ layer and a 1.8‐nm‐thick IGZO interlayer, sandwiched between bottom TiN and top TaN electrodes. The structural integrity of this design was verified through Cs‐corrected scanning transmission electron microscopy (STEM) and corresponding energy‐dispersive X‐ray spectroscopy (EDS) elemental mapping (Figure 3a–c and Figure S4). The TiN and TaN electrodes were intentionally selected to exploit their work‐function difference (∼0.29 eV; Figure S5), enhancing tunneling‐barrier modulation during polarization charging and relaxation. For the IGZO interlayer, we prepared three distinct atomic compositions with In:Ga:Zn ratios of 1:1:1, 2:2:1, and 2:1:1. These variants are hereafter referred to as 1:1:1‐ZrO_2_, 2:2:1‐ZrO_2_, and 2:1:1‐ZrO_2_ AFTJs. These compositional variations were validated by energy‐dispersive X‐ray fluorescence (EDXRF) and by comparing the relative intensities in time‐of‐flight secondary ion mass spectrometry (ToF‐SIMS) measurements (Figure 3d–f and Figure S6). Details of the fabrication process and material characterization equipment are provided in Section 4.

**FIGURE 4:**
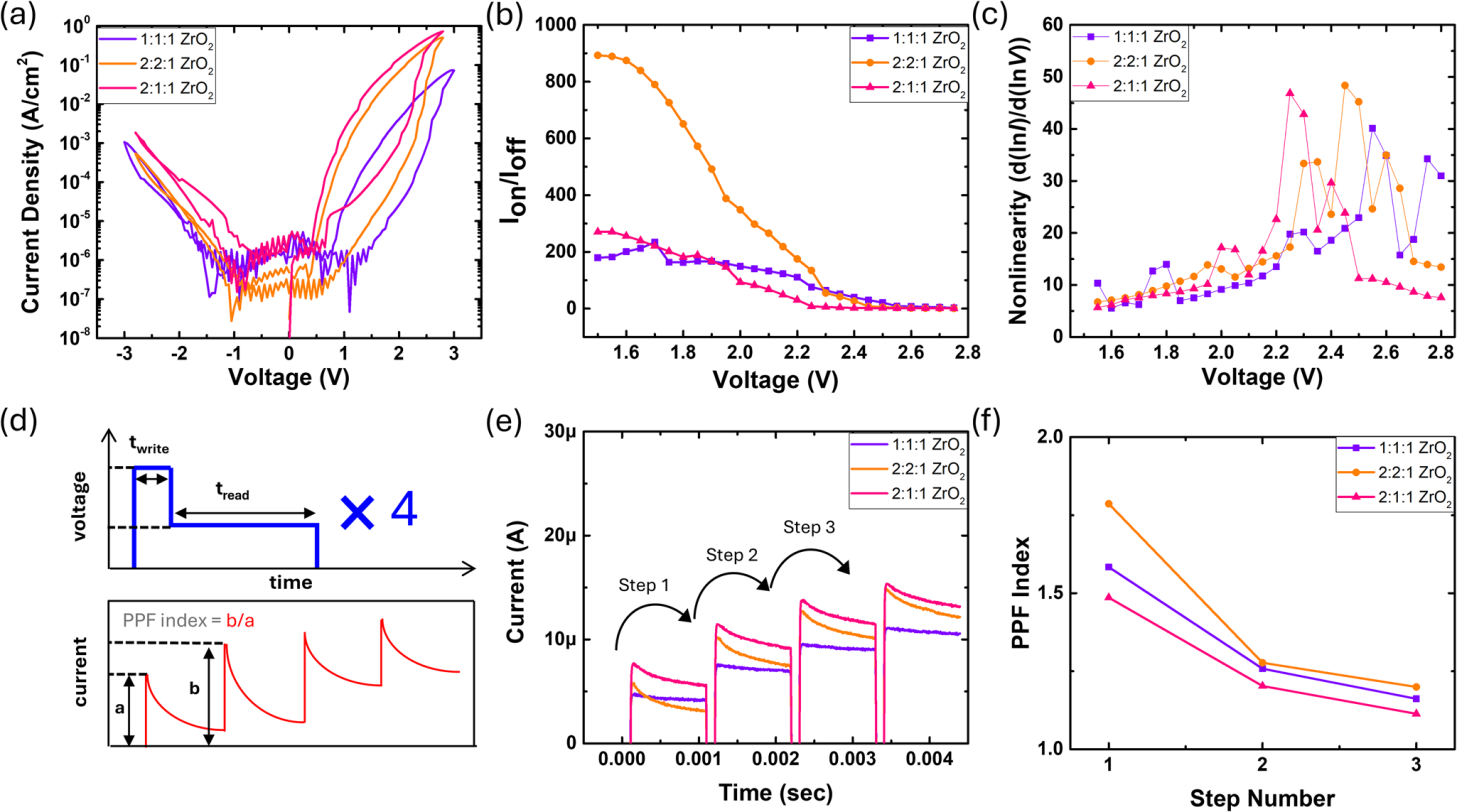
Static electrical properties and dynamic STM characteristics of the engineered AFTJs as a function of the a‐IGZO stoichiometry. (a) Representative DC current density–voltage (*J*–*V*) hysteresis curves of the 1:1:1, 2:2:1, and 2:1:1‐ZrO_2_ AFTJs, where the numerical ratios denote the In:Ga:Zn atomic ratio of the a‐IGZO interlayer. (b) Extracted memory margins (I_on_/I_off_) and (c) calculated nonlinearity factors (NL), defined as d(ln I)/d(ln V), plotted as a function of the applied voltage. (d) Schematic illustration of the dynamic measurement protocol using a four‐step sequential read–write paired‐pulse train (top) and the definition of the PPF index derived from the transient decay currents (bottom). (e) Transient current responses recorded during the successive read pulse intervals for the three compositional variants. (f) Calculated PPF indices corresponding to each sequential step.

We acquired DC current density‐voltage (*J*–*V*) sweep curves and transient current responses under four sequential paired read–write pulses for the three compositional variants. Prior to these single‐device measurements, we characterized the evolution of the device characteristics under ±3 V voltage pulses at 50 kHz. This wake‐up procedure was implemented to stabilize the structural phase and suppress any subsequent performance variations arising from initial electric‐field cycling in the ZrO_2_ layer. Following this preconditioning, all single‐device performance parameters were collected after subjecting each AFTJ device to 10^4^ electric‐field pulses (see Figure S7 for wake‐up and fatigue processes of the devices). As plotted in Figure 4a, the applied DC voltage for I‐V measurements was swept from 0 to 2.9 V, then to −2.9 V, and finally returned to 0 V with a step size of 0.05 V. Although the full sweep range was recorded, we excluded the low‐voltage regions (<1.5 V) for the subsequent parameter extraction, where the sensing current was heavily dominated by background noise near 0 V.

From the *I*–*V* characteristics, we established two key quantitative electrical parameters to benchmark the performance of the reservoir neurons: the I_on_/I_off_, which represents the accessible dynamic range, and the nonlinearity factor (NL), defined as d(ln I)/d(ln V). The extracted I_on_/I_off_ ratios exhibit a clear dependence on the IGZO stoichiometry (Figure 4b). Specifically, the 2:2:1‐ZrO_2_ AFTJ achieves the highest I_on_/I_off_ of ∼890, significantly outperforming both the 1:1:1‐ZrO_2_ (∼180) and the 2:1:1‐ZrO_2_ (∼270) devices. Alongside the memory margin, the nonlinearity factor (NL) of the current response—a critical parameter for determining the computational richness of a physical reservoir—exhibits a similar compositional dependence (Figure 4c). When evaluated within the relevant operating voltage window of 1.5–2.9 V, the 2:2:1‐ZrO_2_ device demonstrates the most pronounced nonlinear behavior, achieving a maximum NL of ∼48.4 and an average NL of ∼18.6. In comparison, the 1:1:1 and 2:1:1 compositions yield lower average NL values of ∼16.4 and ∼14.9, respectively.

The maxima at 2:2:1 can be attributed to the balance between In‐driven carrier generation and Ga‐driven leakage suppression [48, 49, 50]. As the In content increases from 1:1:1 to 2:2:1, the overall current level rises, consistent with In increasing the free‐carrier density through shallow donor states and s‐orbital overlap. These intrinsic characteristics enhance the on‐state tunneling current, contributing to a steeper and nonlinear current onset [49, 51, 52]. However, as the In ratio becomes excessive in the 2:1:1 composition, this advantage is outweighed by a severe degradation in both off‐current suppression and tunneling nonlinearity. According to the defect models reported for IGZO systems, Ga acts as a crucial carrier suppressor due to its strong oxygen‐binding energy [52, 53]. In the In‐rich 2:1:1 stoichiometry, the deficiency of Ga fails to suppress the formation of oxygen vacancies and undercoordinated metal atoms. As reported in the literature, these defects introduce electronic trap states within the bandgap, creating unwanted hopping conduction pathways [54, 55]. Trap‐assisted hopping increases leakage and linearizes transport, reducing AFTJ nonlinearity.

Consequently, the 2:2:1 composition represents an optimal balance between In‐driven carrier generation and Ga‐driven leakage suppression, yielding both the maximum dynamic range and the highest nonlinearity.

To evaluate the impact of static parameters on the dynamic performance, we investigated the STM characteristics across the engineered AFTJs. Figure 4d illustrates the measurement scheme using a four‐sequential write‐read paired pulse train alongside the definition of the paired‐pulse facilitation (PPF) index, a standard metric for quantifying STM behavior. Each unit sequence consists of a 3 V, 100 µs write pulse followed by a 1.8 V, 1 ms read pulse, repeated four times. The resulting transient current responses during the read pulses and their corresponding calculated PPF indices are presented in Figure 4e,f, respectively.

The transient measurements reveal a distinct correlation between the static switching performance and the dynamic neuromorphic capabilities of the AFTJs. All compositions maintain PPF indices above one, confirming their fundamental viability as functional PRC neurons. However, the magnitude of temporal integration varies with the IGZO stoichiometry. The 2:2:1‐ZrO_2_ AFTJ demonstrates the most robust history‐dependent integration, yielding the highest initial PPF index of ∼1.79. In contrast, the 1:1:1‐ZrO_2_ and 2:1:1‐ZrO_2_ devices exhibit weaker temporal accumulation, with maximum initial PPF indices of ∼1.58 and ∼1.49, respectively (see Figure 4f). To further verify whether this single‐device STM trend is retained at the reservoir level, we additionally evaluated the linear memory capacity of the ZrO_2_‐based AFTJ reservoirs using random input sequences and delayed‐input reconstruction; the detailed definition, protocol, and analysis are provided in Figure S8 [16, 22]. Consistent with the PPF trend, the optimized 2:2:1‐ZrO_2_ AFTJ exhibits the highest total memory capacity among the compared IGZO stoichiometries, confirming that its transient states preserve past‐input information more effectively.

Although both I_on_/I_off_ and NL peak at the intermediate In content (2:2:1), their compositional hierarchies differ; the PPF index follows the NL trend rather than the I_on_/I_off_ trend. Specifically, while the I_on_/I_off_ follows a hierarchy of (2:2:1 > 2:1:1 > 1:1:1), the PPF and average NL values follow the same distinct order: the PPF index (1.79 > 1.58 > 1.49) mirrors the compositional hierarchy of the average NL evaluated across the operating voltage window (18.6 > 16.4 > 14.9 for the 2:2:1, 1:1:1, and 2:1:1 compositions, respectively). This indicates that the dynamic fading memory characteristics of the reservoir node, quantified by the PPF index, exhibit a stronger correlation with the inherent current nonlinearity rather than the static memory margin.

The 2:2:1 stoichiometry, which achieved the highest tunneling nonlinearity by optimally balancing carrier generation and leakage suppression, also exhibits the strongest temporal signal integration among the three compositions. This result suggests that the PPF response is not determined by the absolute current level or static memory margin alone, but is also governed by the presence of a nonlinear read‐current modulation pathway that converts the volatile AFE polarization state into a distinguishable transient current response. The 2:1:1 device, in which the In‐driven current enhancement is no longer compensated by the Ga‐mediated leakage suppression, is consistent with this interpretation, showing a larger overall current level together with a reduced NL and a lower PPF response. The static and room‐temperature pulse measurements alone, however, do not separate two possible contributions to the observed tunnel‐current modulation: the volatile AFE polarization switching of the ZrO_2_ layer and the interfacial charge/trap dynamics at the IGZO/ZrO_2_ interface. In particular, it is not established from these measurements alone whether the role of the IGZO interlayer is confined to enlarging the memory margin or also extends to the transient relaxation kinetics that govern the fading response in Figure 4e.

To examine these candidate contributions, we performed a temperature‐dependent transient measurement on the optimized 2:2:1‐ZrO_2_ AFTJ from 300 to 380 K (Figure S9). The two candidate processes are expected to differ in their Arrhenius behavior: volatile AFE back‐switching, governed by a small phase‐transition barrier, would exhibit only a weak thermal dependence, whereas trap‐assisted transport through the IGZO/ZrO_2_ interface would show a stronger temperature dependence. The post‐peak relaxation time constant exhibits a weak thermal activation comparable in magnitude to the kinetic barriers reported for field‐induced antiferroelectric phase transitions in HZO, whereas the decay amplitude and the asymptotic read current follow Arrhenius dependences with activation energies roughly two to four times larger, consistent with thermally activated trap‐mediated conduction in the IGZO interlayer. The slow relaxation time scale is also more than an order of magnitude longer than the measurement charging time, excluding capacitive discharge as its origin. Taken together (full quantitative analysis in Figure S9 and accompanying discussion), the resolved post‐peak transient is interpreted as a stack‐coupled response in which the slow back‐relaxation of the field‐induced o‐phase polarization in the ZrO_2_ layer sets the fading time scale and modulates the effective tunnel barrier at the ZrO_2_/IGZO interface, while the long‐time read‐current magnitude is governed by trap‐mediated conduction through the IGZO interlayer. The IGZO interlayer therefore assumes a dual role: it sets the read‐current magnitude through its trap‐mediated conductance and contributes to the long‐time activation energy, while the resolved sub‐millisecond relaxation timescale carries the imprint of the underlying AFE polarization dynamics in the ZrO_2_ layer.

These results collectively indicate that a high memory margin is necessary to secure a wide dynamic range, that the sub‐millisecond fading response of the optimized 2:2:1‐ZrO_2_ AFTJ originates from a stack‐coupled transient in which the slow AFE back‐relaxation of the ZrO_2_ layer dominantly sets the resolved time scale and the IGZO interlayer registers this barrier modulation as a time‐varying trap‐mediated current, and that efficient temporal integration in AFTJ reservoir neurons benefits from nonlinear current modulation combined with controlled off‐current and leakage pathways at the IGZO/ZrO_2_ interface.

### 2.3 Multibit Memory Performance of Reservoir Neurons and Digit Recognition

Static and STM metrics alone do not guarantee robust reservoir performance. Because reservoir operation relies on its transient temporal states to encode time‐varying inputs, the effectiveness of the neuron must be evaluated by its state richness and distinguishability across the time domain. Thus, we introduced two integrated metrics—*bit capacity* and *state distinguishability*—to connect material properties with multibit signal processing in recognition tasks.

Before detailing the multibit representation, it is crucial to address the device endurance constraints. As previously shown in Figure S7, the high on‐current in the 2:1:1‐ZrO_2_ AFTJ caused severe degradation, resulting in an endurance of only ∼10^6^ cycles, approximately one order of magnitude lower than that of the other devices (>10^7^ cycles). Thus, 2:1:1‐ZrO_2_ was excluded from multibit tests and replaced by 2:2:1‐Hf_0.2_Zr_0.8_O_2_ for comparison.

**FIGURE 5:**
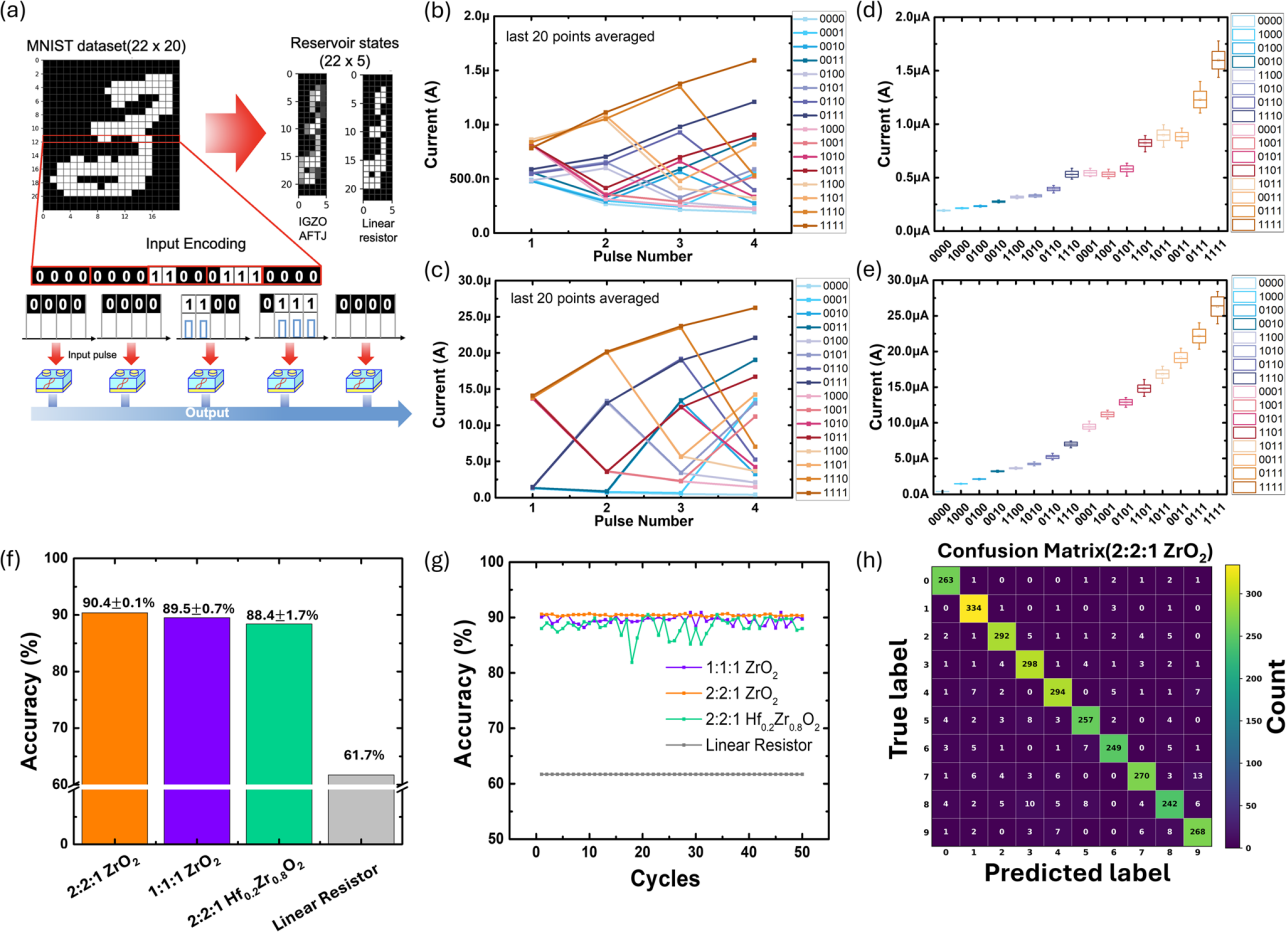
Multi‐bit state distinguishability of AFTJ devices and corresponding evaluation of spatiotemporal data compression for the MNIST digit recognition task. (a) Schematic overview of the image data encoding process. High‐dimensional spatial images (22 × 20 pixels) are sequentially encoded into 4‐bit input pulses and mapped onto the multibit transient states of the AFTJ reservoir, effectively compressing the dimensionality to a 22 × 5 state matrix. (b, c) Transient current trajectories corresponding to 16 distinct 4‐bit input patterns (from “0000” to “1111”) for the (b) 2:2:1(In:Ga:Zn)‐Hf_0.2_Zr_0.8_O_2_ and (c) optimal 2:2:1‐ZrO_2_ AFTJs. (d, e) Statistical distributions of the extracted memory state currents (averaged over the final 20% of the read pulse) collected over 50 consecutive cycles for the (d) 2:2:1‐Hf_0.2_Zr_0.8_O_2_ and (e) 2:2:1‐ZrO_2_ devices. (f) Overall MNIST classification accuracy of the engineered AFTJ reservoirs compared with a simple linear resistor baseline. (g) Cycle‐to‐cycle recognition accuracy over 50 epochs. (h) Confusion matrix for the 2:2:1‐ZrO_2_ AFTJ. The numerical ratios denote the In:Ga:Zn atomic ratio of the a‐IGZO interlayer.

The scheme for utilizing the multibit transient states of the AFTJ for MNIST dataset classification is illustrated in Figure 5a, while more details are included in Note S3. To quantify the *bit capacity*, we applied a customized paired‐pulse scheme resembling the prior STM evaluation. We defined ‘0’ and ‘1’ inputs using 1.8 V and 3.5 V write pulses (200 µs duration), respectively, followed immediately by a 1.8 V read pulse (2 ms duration) (Figure 5b, inset). By sequentially applying these binary inputs, we investigated the transient current levels corresponding to 16 distinct 4‐bit operations ranging from “0000” to “1111”. The representative transient current responses for the 2:2:1‐Hf_0.2_Zr_0.8_O_2_ and 2:2:1‐ZrO_2_ AFTJs are presented in Figure 5b,c, respectively, where each memory state current was extracted by averaging the final 20% of the read pulse duration. Raw traces are shown in Figure S10.

Figure 5b,c reveal distinct bit‐capacity differences. The 2:2:1‐Hf_0.2_Zr_0.8_O_2_ AFTJ demonstrated a severe overlap in its 4‐bit representation, limiting its practical bit capacity to 3 bits. In contrast, both the 1:1:1‐ZrO_2_ and 2:2:1‐ZrO_2_ AFTJs appeared to resolve all 16 states, indicating a functional 4‐bit capacity. To evaluate the *state distinguishability* statistically, we repeated these 4‐bit operations for 50 consecutive cycles. Figure 5d,e map the cycle‐to‐cycle current distribution for the 2:2:1‐Hf_0.2_Zr_0.8_O_2_ and 2:2:1‐ZrO_2_ devices, respectively, with error bars encompassing 90% of the distribution spread (Figure S10d–f illustrate the current state distributions for all three AFTJs obtained during 50 repetitions of 4‐bit operation, accompanied by the extracted mean and standard deviation data.).

These results reveal clear differences in multibit representation performance among the devices. The 2:2:1‐Hf_0.2_Zr_0.8_O_2_ device is limited to a 3‐bit capacity because severe overlap between nominal 4‐bit states prevents reliable state discrimination during repeated operation (Figure 5d). Such inter‐state overlap limits reliable readout classification. In contrast, both the 1:1:1‐ZrO_2_ and 2:2:1‐ZrO_2_ devices achieve 4‐bit capacities. Furthermore, when evaluating the standard deviation (STD) of each state and the frequency of overlapping data points across 50 operational cycles, the 2:2:1‐ZrO_2_ device (Figure 5e) maintains tightly clustered distributions with a lower probability of overlap, with the worst‐case overlap being 12% for the “1101”—“1011” pair and the mean overlap being only 2.53%, indicating superior state discrimination.

State distinguishability is governed by current‐level separation relative to transient noise and cycle‐to‐cycle vatiability (CtCV), and is therefore more closely linked to the quasi‐static memory margin than to the peak‐current‐based PPF index. Specifically, the restricted bit capacity of the 2:2:1‐Hf_0.2_Zr_0.8_O_2_ device is directly attributed to its relatively low I_on_/I_off_ ratio; the correspondingly narrow memory‐state current levels are easily obscured by fundamental transient current noise.

On the other hand, while both the 1:1:1‐ZrO_2_ and 2:2:1‐ZrO_2_ devices possess sufficient absolute memory‐state current levels to support 4‐bit operation, the 50‐cycle repetition test reveals a distinct difference in their dynamic stability. Benefiting from a larger I_on_/I_off_ ratio, the 2:2:1‐ZrO_2_ device exhibits greater resistance to CtCV. Thus, the 2:2:1‐ZrO_2_ device provides the strongest multibit reservoir performance. These findings confirm that the quasi‐static memory margin, discussed in Section 2.2, is correlated with the enhanced state richness in PRC neuron devices.

To evaluate the impact of this physical state distinguishability on spatiotemporal data compression and hardware area efficiency, we utilized the MNIST digit‐recognition task [56]. MNIST images were segmented into 4‐bit input patterns and mapped onto the 16 device states. The resulting reservoir‐state vectors were classified using a support vector machine. Details are provided in Note S3. As shown in Figure 5f, the classification accuracy is consistent with our state‐distinguishability analysis. While all AFTJ PRC neurons outperform the simple linear resistor baseline (61.7%), which lacks memory of past states, the optimal 2:2:1‐ZrO_2_ AFTJ achieves the highest overall accuracy of 90.4% ± 0.1%, outperforming the 1:1:1‐ZrO_2_ (89.5% ± 0.7%) and the lower capacity 2:2:1‐Hf_0.2_Zr_0.8_O_2_ (88.4% ± 1.7%) devices.

Furthermore, we verified how the CtCV and dynamic state fluctuations impact system‐level performance by evaluating the cycle‐to‐cycle recognition accuracy across the 50 cycles, as shown in Figure 5g. We applied the specific 4‐bit current values extracted from each of the 50 corresponding cycles to perform an independent classification test for each cycle. This dynamic evaluation demonstrates that maintaining a clear separation between individual states is critical. By preventing state overlap, the optimized device maintains high accuracy despite the continuous variability inherent in repeated operations. As demonstrated by mapping the temporal data onto the fluctuating state distributions of each cycle, the optimized 2:2:1‐ZrO_2_ AFTJ mitigated the impact of inherent variability owing to its robust state distinguishability. Consequently, the optimized 2:2:1‐ZrO_2_ device demonstrated operational consistency, maintaining bounded, high‐accuracy performance. By overcoming the limited bit‐capacity of the 2:2:1‐Hf_0.2_Zr_0.8_O_2_ and exhibiting superior state distinguishability compared to the 1:1:1‐ZrO_2_, the optimized device demonstrates strong multibit reservoir performance.

The corresponding confusion matrix for the optimal 2:2:1‐ZrO_2_ device is provided in Figure 5h, confirming its reliable classification across all digits. This high accuracy is achieved even after compressing the original 22 × 20 pixel image into a 22 × 5 pixel reservoir state matrix. We reduced the dimensionality of the input data by mapping spatial pixel data into the temporal domain using the distinct multilevel states of the device. While the simple linear resistor fails to distinguish the compressed patterns due to interstate overlap and the absence of fading memory, the superior state distinguishability of the 2:2:1‐ZrO_2_ AFTJ enables this dimensionality reduction without data loss. This reduces the number of required readout weights and the overall computational cost while maintaining recognition performance. Overall, the large memory margin of the 2:2:1‐ZrO_2_ AFTJ stabilizes multibit transient states against noise and CtCV, enabling 4‐bit encoding and 90.4% MNIST accuracy under compressed spatiotemporal encoding.

### 2.4 Temporal Signal Processing Performance of the AFTJ Reservoir

We next evaluate temporal processing using waveform classification [56, 57], Hénon‐map prediction [58], and real‐world semiconductor‐index forecasting. Furthermore, to compare the proposed AFTJ reservoir within the current technological landscape, we fabricated photolithography‐patterned AFTJs and analyzed their area‐dependent decay characteristics across a device area range from 40 000 to 400 µm^2^. Based on these scaling trends, we estimated the operational speed and energy consumption for a further scaled 100 µm^2^ device, benchmarking these metrics against reported physical reservoir systems.

**FIGURE 6:**
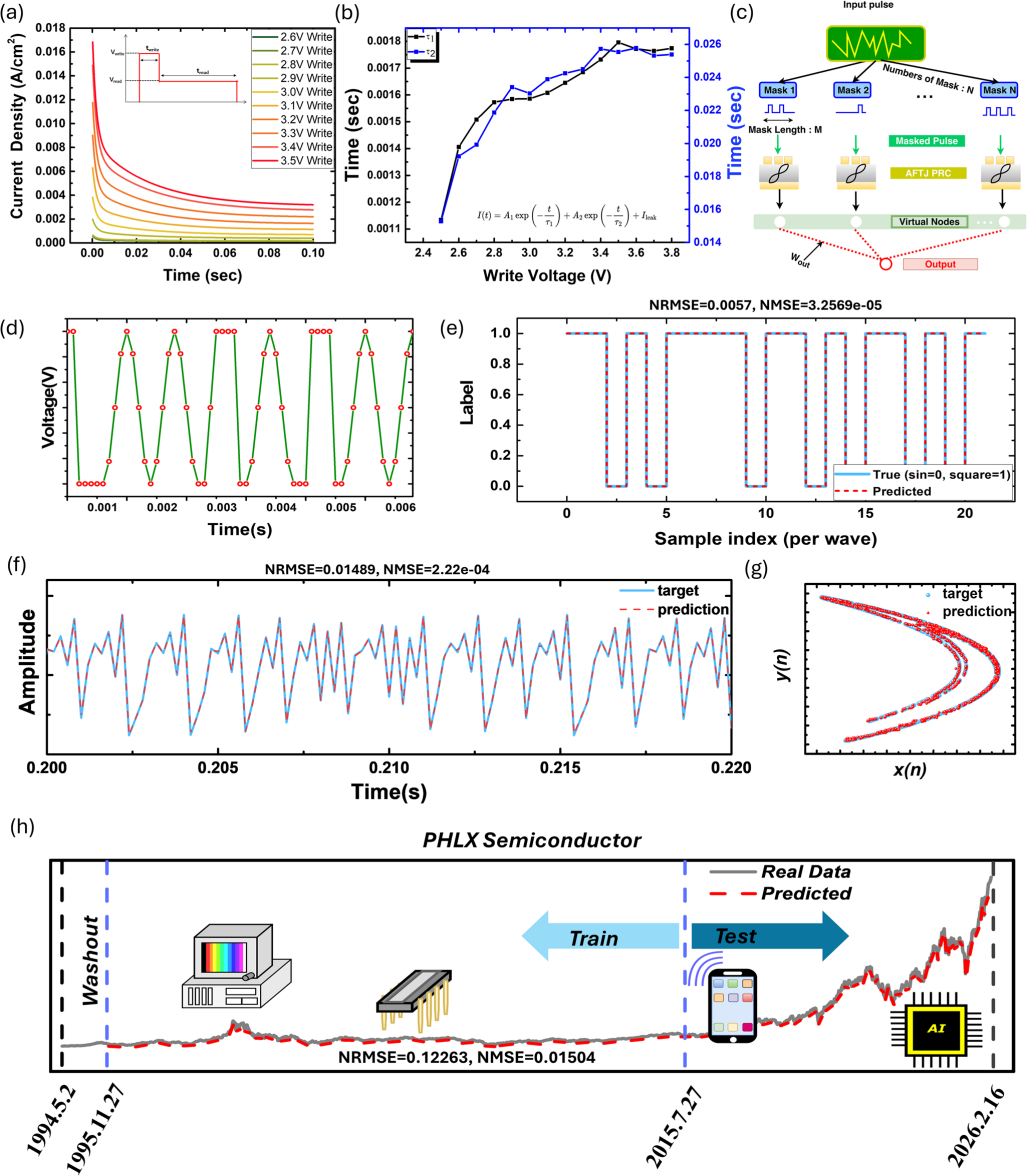
Circuit‐level emulation and evaluation of temporal data processing capabilities using the optimal 2:2:1‐ZrO_2_ AFTJ reservoir. (a) Transient current decay curves of the AFTJ measured under a constant read voltage after applying various write voltages ranging from 2.6 to 3.5 V. The inset details the measurement pulse scheme. (b) Voltage‐dependent decay time constants (τ_1_ and τ_2_) extracted by fitting the empirical relaxation curves in (a) with a double‐exponential function, which were then incorporated into the *ngspice* simulation environment. (c) Schematic illustration of the temporal masking process where continuous input streams are transformed into high‐dimensional transient states (virtual nodes) using the experimentally validated device model. (d) Alternating sinusoidal and square wave input signals used for the waveform classification task, and (e) the corresponding predicted output labels vs. the true labels (NRMSE = 0.0057). (f) One‐step‐ahead prediction results of the chaotic Hénon map. The predicted sequence agrees well with the true target (NRMSE = 0.01489). (g) Reconstructed 2D phase‐space map. (h) Prediction performance on a real‐world financial time‐series dataset using the weekly Philadelphia Semiconductor Index (PHLX SOX) from 1994 to 2026. The testing phase confirms accurate tracking of the actual unstructured index data (NRMSE = 0.12263).

To evaluate the AFTJ in complex time‐series dataset tasks requiring continuous processing of sequential inputs, we established a circuit‐level simulation framework that captures its intrinsic device dynamics. More details are included in Note S3. Because arbitrarily long input sequences are impractical to measure directly, we built an experimentally calibrated circuit‐level model. We characterized the temporal current relaxation of the optimal 2:2:1‐ZrO_2_ AFTJ by monitoring its transient decay across a range of write voltages (from 2.6 to 3.5 V) under a constant read voltage (Figure 6a). By fitting these empirical relaxation curves with a double‐exponential function,

(1) $$I\;\left(t\right)=A_{1}\;exp\left(-\frac{t}{\tau_{1}}\right)+A_{2}\;\exp\left(-\frac{t}{\tau_{2}}\right)+I_{leak})$$

we extracted the voltage‐dependent decay time constants, τ_1_ and τ_2_ (Figure 6b). These parameters were subsequently incorporated into an *ngspice* simulation environment. This modeling approach captures the spontaneous relaxation behavior of the AFTJ, enabling the emulation of natural fading memory without requiring any explicit reset operations. Using this experimentally validated model, continuous input streams were transformed into high‐dimensional transient reservoir states via a temporal masking process, generating a sequence of virtual nodes for subsequent data processing (Figure 6c). Further details regarding the simulation protocol are provided in the Experimental Section.

Using this emulated reservoir, we evaluated a waveform classification task designed to distinguish between sinusoidal and square waves (Figure 6d). Sinusoidal and square inputs had identical amplitude ranges and periods, so classification depended on nonlinear fading‐memory dynamics. In PRC, continuous pulse application over an extended period can cause gradual accumulation of the internal device state and eventual saturation, which degrades the system's ability to accommodate and distinguish new temporal features. To prevent this saturation and maintain the optimal operating state of the device, the input pulses were confined to the range from −3 to 3 V. The phase‐transition dynamics of the AFTJ amplify the differences between the smoothly varying current responses induced by sinusoidal inputs and the abrupt transient shifts generated by square waves. To maximize processing efficiency, the total number of reservoir nodes was fixed at 36, and the temporal partitioning scheme was varied. Under the optimal configuration of a mask length of 4 with 9 virtual nodes, as determined from the masking process comparison summarized in Table S3, the AFTJ reservoir classified the continuous waveforms with low error. As shown in Figure 6e, the predicted output closely matches the true labels, achieving a low normalized root mean square error (NRMSE) of 0.0057 and a normalized mean square error (NMSE) of 3.25 × 10^−5^.

To validate the reservoir's capacity for complex time‐series forecasting, we extended our evaluation to the one‐step‐ahead prediction of a Hénon map. The Hénon map was used as a chaotic one‐step‐ahead prediction benchmark, and is defined by the following relations:

(2) $$x\;\left(k+1\right)=1-1.4x^{2}\left(k\right)+y\left(k\right)$$

(3) $$y\;\left(k+1\right)=0.3\;x\left(k\right)$$

Because forecasting its subsequent state depends on retaining the history of preceding states, this task serves as a benchmark requiring both nonlinearity and STM from the reservoir node. By applying the optimal masking configuration (9 virtual nodes and a mask length of 4) that yielded the lowest error in Table S3 and extending the simulation duration to 0.4 s, the AFTJ reservoir demonstrated accurate tracking of the chaotic signal. Figure 6f illustrates the strong agreement between the predicted sequence and the true Hénon map target, yielding a low NMSE of 2.22 × 10^−4^ (NRMSE = 0.01489). This temporal prediction is further supported by the reconstructed 2D phase‐space mapping (Figure 6g), where the predicted states trace the characteristic strange attractor of the chaotic system.

To further demonstrate the practical applicability of the AFTJ physical reservoir, we evaluated its prediction performance on real‐world financial time‐series datasets as shown in Figure 6h. Specifically, we utilized the weekly Philadelphia Semiconductor Index, known as PHLX SOX, from 1994 to February 2026. The actual index values were linearly mapped to voltage pulse amplitudes ranging from 1.8 to 3.8 V. This mapping range from 1.8 to 3.8 V was selected to prevent the complete decay of internal states below the effective write voltage while securing sufficient dynamic range for the rising data. These input pulses were applied to the experimentally calibrated model of the optimal 2:2:1‐ZrO_2_ AFTJ. To extract the temporal features, we applied a masking process with a mask length of four and nine virtual nodes. The sequential data were divided into three distinct phases. The period from 1994 to November 1995 was discarded as a washout phase to stabilize the initial reservoir states. The subsequent data from December 1995 to July 2015 were used to train the readout weights. Finally, the remaining data from August 2015 to February 2026 were used for the testing phase. The predicted index closely tracks the actual PHLX SOX data, yielding an NMSE of 0.01504 and an NRMSE of 0.12263. These results indicate that the experimentally calibrated AFTJ model captures reservoir dynamics relevant to practical temporal‐processing tasks and supports the use of this device platform for edge computing applications.

**FIGURE 7:**
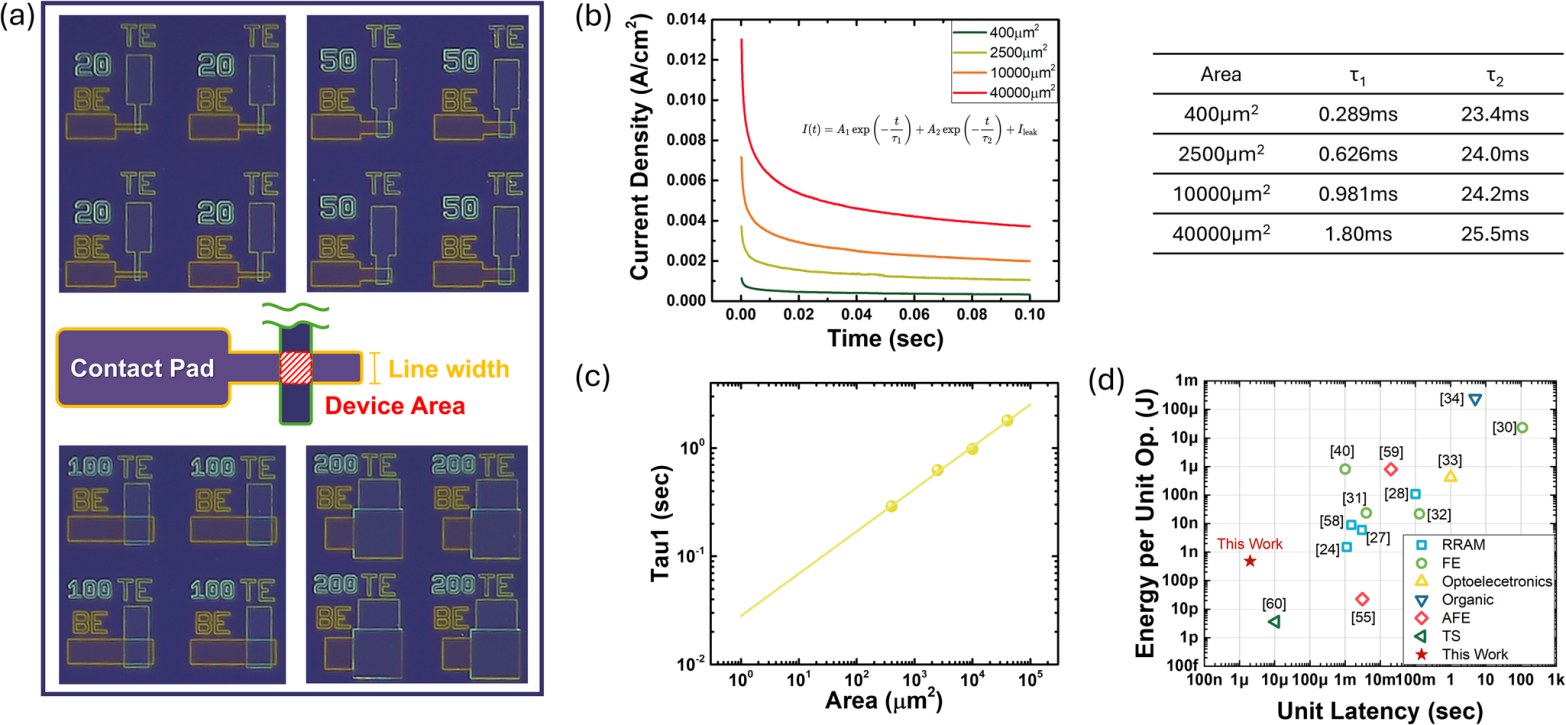
Area scalability, temporal dynamics projection, and performance benchmarking of the patterned AFTJs. (a) Optical microscope images and corresponding schematic of the photolithography‐patterned AFTJs with controlled active device areas ranging from 40 000 down to 400 µm^2^. (b) Area‐dependent transient current relaxation curves following a uniform pulse stimulation. The inset table summarizes the corresponding fast (τ_1_) and slow (τ_2_) decay time constants extracted via double‐exponential fitting. (c) Power‐law fit of τ_1_ vs. device area, (d) Benchmark of unit latency and energy per operation using experimentally demonstrated devices; the projected 100 µm^2^ AFTJ value is indicated separately and is not used for direct ranking.

Beyond computational accuracy, practical edge‐computing hardware must satisfy strict requirements for latency and power consumption. To evaluate these physical metrics and establish a fair benchmark against memristive reservoir systems, we scaled down the device area through a photolithography patterning process. Figure 7a presents an optical microscope image and the corresponding schematic of the patterned AFTJs, designed to control the active device area. Using these scaled devices, we monitored the transient current responses across varied device sizes ranging from 40 000 µm^2^ down to 400 µm^2^ (Figure 7b). The resulting relaxation curves were fitted with the established double‐exponential function to extract the area‐dependent time constants, τ_1_ and τ_2_, as summarized in the adjacent inset table.

The extracted fast decay constant (τ_1_), which dictates the operational speed of the reservoir, exhibits a distinct scaling dependence. In Figure 7c, the experimentally measured (*A*,τ_1_) data were fitted using a power‐law relation, $\log_{10}\tau_{1}=m\log_{10}A+b$, and this fitted trend was used to estimate the latency and energy consumption of a further scaled 100 µm^2^ device. Direct transient characterization of the 100 µm^2^ device at the projected operation window is experimentally challenging because device‐area reduction is expected to decrease the absolute decay current while the relevant pulse and relaxation times are shortened into the sub‐µs regime. In the present measurement configuration, this combination of low‐current readout and sub‐µs operation reduces the signal‐to‐noise margin required for reliable extraction of decay constants, motivating the use of the area‐dependent extrapolation rather than direct time‐series operation of a 100 µm^2^ device.

For the experimentally demonstrated 40 000 µm^2^ AFTJ reservoir, the temporal‐processing pulse scheme consists of a 0.18 µs write pulse and a 1.8 µs read pulse, corresponding to a unit latency of approximately 2 µs. Based on the measured transient current responses under this pulse scheme, the energy consumption is conservatively estimated to be less than 480 pJ per unit operation. Applying the fitted τ_1_‐scaling trend to the same temporal‐processing scheme indicates that a 100 µm^2^ AFTJ could operate with unit pulses approximately one‐tenth the duration of those required for the 40 000 µm^2^ baseline device, yielding a projected unit latency of ∼192 ns. Considering both this shortened operation time and the proportional reduction in absolute current magnitude with device area, the projected energy consumption of the 100 µm^2^ AFTJ is estimated to be <115 fJ per unit operation. A detailed description of the measurement limitations, τ_1_‐based latency extrapolation, and conservative energy calculation procedure is provided in Note S2.

As summarized in Figure 7d and Table S4, these experimentally obtained metrics already place the present AFTJ among the most competitive physical reservoir devices reported to date, showing the fastest operational speed and the third‐lowest energy dissipation among the benchmarked systems. The projected 100 µm^2^ value should therefore be interpreted as an analytical scaling projection rather than a directly measured benchmark. This extrapolation assumes that the power‐law dependence of τ_1_, extracted from the experimentally measured 40 000–400 µm^2^ devices, remains valid down to 100 µm^2^ and that the absolute current scales approximately with the active device area. Possible deviations caused by edge leakage, process‐dependent variability, parasitic RC components, measurement bandwidth, and array‐level interconnect overhead are not included in the present projection. Therefore, the experimentally fabricated 40 000 µm^2^ AFTJ is used for direct benchmarking, whereas the 100 µm^2^ value is presented only to indicate the potential scaling benefit of further device miniaturization. Compared with previously reported HZO‐based reservoirs, the present AFTJ emphasizes volatile antiferroelectric self‐relaxation in a two‐terminal stack rather than nonvolatile ferroelectric state retention or gated transistor operation. This device‐level difference directly links the material relaxation process to the reset‐free fading‐memory dynamics required for high‐speed PRC. Consequently, the engineered AFTJ reservoir simultaneously delivers accurate temporal data processing capability and promising energy‐speed characteristics for hardware‐oriented edge‐computing applications.

## 3 Conclusion

In summary, volatile AFTJs based on antiferroelectric ZrO_2_ provide a reset‐free platform for rapid PRC. The field‐induced tetragonal‐to‐orthorhombic transition and spontaneous back‐switching generate the combination of nonlinear response and fading memory required for temporal information processing within the ms regime. Incorporation of an ultrathin IGZO interlayer expands the accessible current window, and stoichiometric tuning identifies the 2:2:1‐ZrO_2_ stack as the most balanced design. This device delivers the largest Ion/Ioff and the strongest nonlinear current response among the tested compositions, together with the highest PPF index. The data further suggest that temporal integration correlates more closely with current nonlinearity, whereas multistate separability benefits from a large memory margin. Consistent with this picture, the 2:2:1‐ZrO_2_ AFTJ exhibits the most reliable 4‐bit state representation and reaches 90.4% MNIST accuracy under compressed spatiotemporal encoding.

Temporal tasks were evaluated using a circuit‐level reservoir model calibrated with measured device relaxation curves. The AFTJ reservoir performs waveform classification, one‐step‐ahead Hénon prediction, and forecasting of a representative noisy, real‐world semiconductor‐index time‐series dataset. The fabricated 40 000 µm^2^ device demonstrates ∼2 µs unit latency and energy consumption below 480 pJ per operation. Projected values for smaller 100 µm^2^ devices, derived from area‐dependent decay trends, indicate further improvement through scaling; however, the projected ∼192 ns latency and ∼115 fJ per operation should be regarded as analytical estimates rather than direct benchmark results. Overall, ZrO_2_‐based AFTJs constitute a promising material/device platform for fast, energy‐efficient, and reset‐free RC hardware.

## 4 Experimental Section/Methods

### 4.1 Sample Fabrication Process

The integrated AFTJ devices and control capacitors were fabricated on heavily doped Si substrates. Initially, a 50‐nm‐thick TiN bottom electrode was deposited via radio‐frequency (RF) sputtering under an argon atmosphere (20 sccm, 1 mTorr). Subsequently, a 6‐nm‐thick dielectric layer of either (Hf,Zr)O_2_ or pure ZrO_2_ was grown by thermal atomic layer deposition (ALD). Tetrakis(ethylmethylamino)hafnium (TEMA‐Hf), tetrakis(ethylmethylamino)zirconium (TEMA‐Zr), and ozone (O_3_) were used as the metal precursors and oxidant, respectively. The precursor sub‐cycles were modulated to yield specific atomic compositions: Hf_0.5_Zr_0.5_O_2_, Hf_0.2_Zr_0.8_O_2_, and pure ZrO_2_. Following deposition, rapid thermal annealing (RTA) was performed at 500°C for 30 s in a N_2_ ambient to induce crystallization and stabilize the requisite antiferroelectric phase. to engineer the asymmetric tunneling barrier, an ultrathin (∼1.8 nm) amorphous In‐Ga‐Zn‐O (a‐IGZO) channel layer was deposited on the crystallized dielectric surface via thermal ALD. Diethylaminoethanol‐indium (DADI), trimethylgallium (TMGa), diethylzinc (DEZ), and O_3_ were employed for the In, Ga, and Zn deposition cycles, respectively. The stoichiometry of the IGZO interlayer was tuned by varying the pulse cycle ratios to obtain the desired atomic compositions, denoted as 1:1:1, 2:1:1, and 2:2:1. Finally, the top electrode was formed by depositing a 50‐nm‐thick TaN layer using direct current (DC) reactive sputtering in an Ar/N_2_ mixed gas environment (10/2 sccm, 5 mTorr). The asymmetric TaN/a‐IGZO/AFE/TiN stack was used to exploit the TiN/TaN work‐function difference.

### 4.2 Material & Chemical Analysis Methods

The crystallographic properties and phase evolution of the engineered (Hf,Zr)O_2_ films were characterized by grazing incidence X‐ray diffraction (GIXRD) using a high‐resolution X‐ray diffractometer (D8 DISCOVER, Bruker) equipped with Cu Kα radiation (λ = 1.5406 Å) at the Research Institute of Advanced Materials (RIAM), Seoul National University. Cross‐sectional structure and layer thicknesses were examined by Cs‐corrected STEM. The STEM imaging was performed using a JEM‐ARM200F (JEOL) operated at an accelerating voltage of 200 kV at RIAM. EDS mapping was performed during STEM analysis for spatial chemical analysis.

Furthermore, the specific atomic compositions and stoichiometric variations of the AFE layers and the a‐IGZO interlayers were quantitatively validated using energy‐dispersive X‐ray fluorescence (EDXRF) and time‐of‐flight secondary ion mass spectrometry (ToF‐SIMS) depth profiling. The ToF‐SIMS measurements were carried out at the National Center for Inter‐university Research Facilities (NCIRF), Seoul National University, utilizing a TOF.SIMS 5 system (ION‐TOF GmbH) equipped with a Bi^+^ primary ion beam to achieve high‐sensitivity compositional depth tracing.

Finally, the work functions of the nitride electrodes (TiN and TaN) were determined using ultraviolet photoelectron spectroscopy (UPS) at NCIRF (AXIS Supra, Kratos Analytical) to establish the built‐in potential difference that governs the asymmetric tunneling behavior. During the UPS measurements, a He I gas discharge lamp was employed as the ultraviolet source, providing photon energy (*h*ν) of 21.22 eV. The absolute work function (Φ) of each electrode was calculated based on the standard photoelectric equation, Φ = hν − (SECO − E_F_), where SECO represents the binding energy of the secondary electron cutoff and E_F_ denotes the Fermi level. Work functions were extracted from the secondary electron cutoff (SECO) using Φ = hν − (SECO − E_F_).

### 4.3 Electrical Characterization of Ferroelectric/Antiferroelectric Capacitors and AFTJs

All electrical evaluations of the capacitors and AFTJ devices were performed under ambient conditions using a Keithley 4200‐A Semiconductor Characterization System (SCS). To verify the intrinsic ferroelectric or antiferroelectric phase evolution, the polarization–voltage (P‐V) hysteresis curves and transient switching current peaks were measured by applying a 3 V, 1 kHz triangular voltage waveform across the devices using the pulse measure units (PMUs). The static current density–voltage (*J*–*V*) hysteresis and tunneling electroresistance properties were measured via continuous DC voltage sweeps using the source‐measure units (SMUs). Before characterization, devices were preconditioned by 10^4^ bipolar pulses at an amplitude of ± 3 V and a frequency of 50 kHz using PMUs. Furthermore, to assess the long‐term reliability and operational stability of the AFTJ devices, endurance cycling tests were performed under 10^0^–10^7^ cycles of ± 3 V rectangular pulses at 50 kHz.

For the dynamic temporal assessments essential for PRC, including STM and multibit performance tests, customized sequential pulse schemes were utilized. Transient decay currents were measured using sequential write/read pulse trains (e.g., 3 V, 100 or 200 µs write / 1.8 V, 1 or 2 ms read). The paired‐pulse facilitation (PPF) indices and cycle‐to‐cycle current variations were subsequently derived by capturing and analyzing the continuous temporal current responses across these consecutive pulse intervals.

### 4.4 Experimental Procedure and Task Configuration

To evaluate the computational capability of the AFTJ‐based PRC system, four representative benchmark tasks were examined. The benchmark tasks included MNIST classification, waveform classification, Hénon‐map prediction, and PHLX SOX forecasting. In all cases, reservoir states were obtained from the current responses of the AFTJ device, and only the readout layer was trained in accordance with the standard PRC framework. Detailed task configurations are provided in Note S3.

### 4.5 Device Modeling and Circuit‐Level Simulation

The measured AFTJ relaxation curves were fitted with a double‐exponential decay model and implemented in ngspice to emulate time‐dependent current responses under masked input pulses. The model allowed each input pulse to accumulate on the residual device state without explicit reset, thereby reproducing the fading‐memory behavior of the AFTJ. Reservoir states for the temporal tasks were obtained from these simulated current responses. Detailed modeling and task protocols are provided in Note S3.

### 4.6 Performance Metrics

Prediction and classification performance was evaluated using the normalized mean square error and the normalized root mean square error. These metrics are defined as follows [27].

(4) $$NMSE=\frac{\sum_{k=1}^{T}{\left[y_{target}\left(k\right)-y\left(k\right)\right]}^{2}}{T\cdot \sigma^{2}\left(y_{target}\right)}\;$$

(5) $$NRMSE=\sqrt{\frac{\sum_{k=1}^{T}{\left[y_{target}\left(k\right)-y\left(k\right)\right]}^{2}}{T\cdot \sigma^{2}\left(y_{target}\right)}}\;$$

Here, *T* denotes the data length in the test phase, and σ^2^(*y_target_*) represents the variance of the target sequence. NMSE quantifies the normalized mean square deviation between the predicted output and the target signal, while NRMSE represents its square root. The prediction performance of the Hénon map task was evaluated using NRMSE, while classification performance was assessed based on the readout outputs using accuracy.

## Author Contributions

**Dong Hee Han**: data curation, investigation. **Taegyu Kwon**: conceptualization, methodology, investigation, validation, writing – original draft. **Ju Yong Park**: data curation, investigation. **Seungho Baek**: data curation, investigation. **Dong Hyun Lee**: writing – review and editing, supervision. **Min Hyuk Park**: conceptualization, writing – review and editing, funding acquisition, supervision. **Moonseek Jeong**: conceptualization, methodology, investigation, validation, writing – original draft. **Su In Hwang**: data curation, investigation. **Joonyong Kim**: data curation, investigation. **Hyeong Seok Choi**: data curation, investigation. **Geun Hyeong Park**: data curation, investigation. **Jung Ho Yoon**: supervision, writing – review and editing. **Da Hyun Kim**: data curation, investigation. **Hyojun Choi**: data curation, investigation.

## Funding

Korean Ministry of Science and ICT (Grant Nos. RS‐2024‐00406897, RS‐2025‐02654040).

## Conflicts of Interest

The authors declare no conflicts of interest.

## Supporting information




**Supporting File**: advs76692‐sup‐0001‐SuppMat.docx.

## Data Availability

The data that support the findings of this study are available from the corresponding author upon reasonable request.
