# Supplementary material for: Volatile ZrO2 Antiferroelectric Tunnel Junctions for Rapid, Energy‐Efficient Physical Reservoir Computing
Source: Adv Sci (Weinh). 2026 Jul 23:e76692. Online ahead of print. doi: 10.1002/advs.76692 (PMC13393266; doi:10.1002/advs.76692)
Supplement: Supplementary file 1 — Supporting File: advs76692‐sup‐0001‐SuppMat.docx. [file ADVS-9999-e76692-s001.docx]

Supporting Information

Volatile ZrO_2_ Antiferroelectric Tunnel Junctions for Rapid, Energy-Efficient Physical Reservoir Computing

Taegyu Kwon^+^, Moonseek Jeong^+^, Su In Hwang, Geun Hyeong Park, Joonyong Kim, Hyojun Choi, Hyeong Seok Choi, Da Hyun Kim, Dong Hee Han, Ju Yong Park, Seungho Baek, Jung Ho Yoon*, Dong Hyun Lee* and Min Hyuk Park*


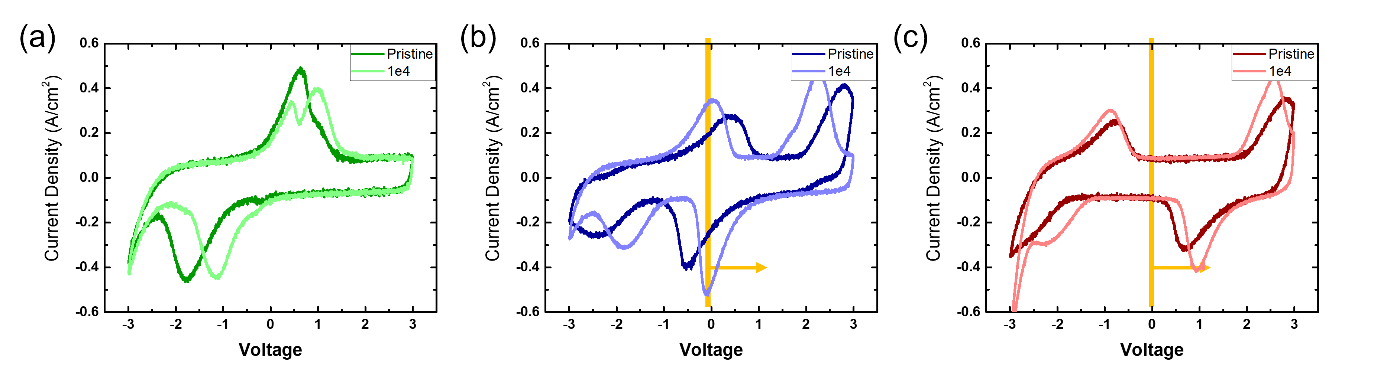


**Figure S1.** **Evolution of the current density–voltage (J-V) hysteresis loops depending on the (Hf,Zr)O_2_ composition.** Transient switching current curves of the **(a)** Hf_0.5_Zr_0.5_O_2_, **(b)** Hf_0.2_Zr_0.8_O_2_, and **(c)** pure ZrO_2_ capacitors evaluated under a 1 kHz triangular voltage waveform with an amplitude of 3 V. The measurements were recorded both in their pristine states (dark lines) and after a preconditioning wake-up process of 10^4^ cycles using 3 V, 50 kHz rectangular pulses (light lines). As the composition becomes more Zr-rich, the AFE-like double switching current peaks become progressively more pronounced. The systematic evolution of the back-switching peak in the positive voltage region highlights the phase differences: after 10^4^ cycles, the back-switching peak of Hf_0.2_Zr_0.8_O_2_ is located near 0 V, indicative of a morphotropic phase boundary (MPB)-like character. Conversely, the back-switching peak of pure ZrO_2_ is located at approximately 1.0 V (yellow arrows), demonstrating a more complete and stabilized AFE response.


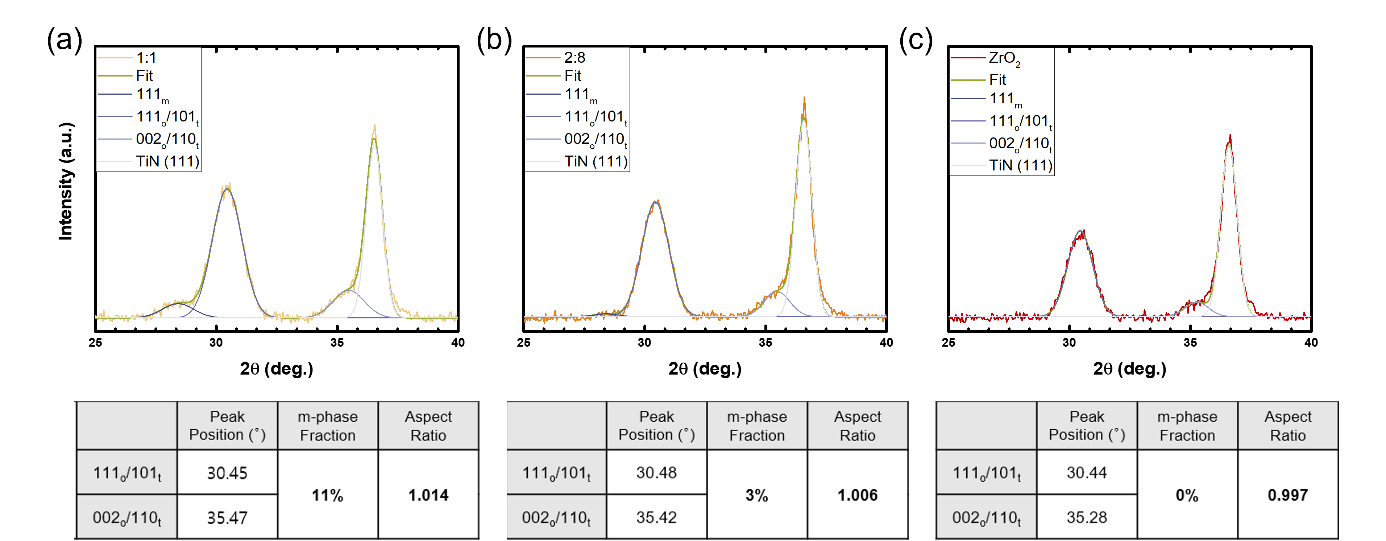


| **Peak Index** | **Peak Type** | **Area Intg** | **FWHM** | **Max Height** | **Center Grvty** | **Area IntgP** |
| --- | --- | --- | --- | --- | --- | --- |
| - 111m | Gaussian | 172.52 | 1.54 | 105.15 | 28.41 | 5.65 |
| 111o/101t | Gaussian | 1458.48 | 1.44 | 950.36 | 30.44 | 47.81 |
| 002o/110t | Gaussian | 328.81 | 1.51 | 203.45 | 35.47 | 10.77 |
| TIN 111 | Gaussian | 1090.44 | 0.80 | 1270.49 | 36.53 | 35.74 |

| **Peak Index** | **Peak Type** | **Area Intg** | **FWHM** | **Max Height** | **Center Grvty** | **Area IntgP** |
| --- | --- | --- | --- | --- | --- | --- |
| - 111m | Gaussian | 28.57 | 1.21 | 22.12 | 28.40 | 1.06 |
| 111o/101t | Gaussian | 1185.68 | 1.31 | 845.76 | 30.48 | 44.18 |
| 002o/110t | Gaussian | 249.65 | 1.24 | 187.63 | 35.41 | 9.30 |
| TIN 111 | Gaussian | 1219.83 | 0.79 | 1445.68 | 36.57 | 45.45 |

| **Peak Index** | **Peak Type** | **Area Intg** | **FWHM** | **Max Height** | **Center Grvty** | **Area IntgP** |
| --- | --- | --- | --- | --- | --- | --- |
| 111o/101t | Gaussian | 815.13 | 1.21 | 632.45 | 30.44 | 40.49 |
| 002o/110t | Gaussian | 131.85 | 1.14 | 108.17 | 35.25 | 6.55 |
| TIN 111 | Gaussian | 1065.74 | 0.79 | 1261.75 | 36.59 | 52.94 |

**Figure S2. GIXRD peak deconvolution analysis of the (Hf,Zr)O_2_ (HZO) films with different Zr contents.** Deconvoluted grazing-incidence X-ray diffraction spectra of the top-electrode-free capacitor stacks for **(a)** Hf_0.5_Zr_0.5_O_2_, **(b)** Hf_0.2_Zr_0.8_O_2_, and **(c)** pure ZrO_2_, showing the fitted contributions from the ${111}_{m}$, ${111}_{o}/{101}_{t}$, and ${002}_{o}/{110}_{t}$ reflections. As the Zr content increases, the spectral weight associated with the monoclinic component is progressively reduced, while the overlapping $o/t$-related reflections become increasingly dominant, indicating a systematic structural evolution toward a t-phase-rich constitution. Consistently, the monoclinic phase fraction estimated from the integrated intensity areal ratio $A_{{111}_{m}}/(A_{{111}_{m}}+A_{{111}_{o}/{101}_{t}}+A_{{002}_{o}/{110}_{t}})$ decreases from 11% for Hf_0.5_Zr_0.5_O_2_ to 3% for Hf_0.2_Zr_0.8_O_2_ and reaches 0% for pure ZrO_2_ (Table S1). The same trend is also reflected in the lattice-derived structural parameters extracted from the fitted peak positions. The aspect ratio, calculated from the ${111}_{o}/{101}_{t}$ and ${002}_{o}/{110}_{t}$ peaks, decreases monotonically from 1.014 to 1.006 and then to 0.997 with increasing Zr content, while the corresponding unit-cell volume decreases from 133.02 to 131.50 and 130.85 Å^3^ for Hf_0.5_Zr_0.5_O_2_, Hf_0.2_Zr_0.8_O_2_, and pure ZrO_2_, respectively (Tables S1 and S2). A detailed procedure for calculating the aspect ratio based on the peak deconvolution analysis is provided in Note S1. These quantitative trends support the progressive suppression of the monoclinic phase and the stabilization of a more compact t-phase-dominant lattice in the Zr-rich regime.

| Sample | Peak index | Peak Position (°) | m-phase fraction | Aspect ratio |
| --- | --- | --- | --- | --- |
| Zr 50% | 111_o_/101_t_ | 30.45 | 11% | 1.014 |
|  | 002_o_/110_t_ | 35.47 |  |  |
| Zr 80% | 111_o_/101_t_ | 30.48 | 3% | 1.006 |
|  | 002_o_/110_t_ | 35.42 |  |  |
| Zr 100% | 111_o_/101_t_ | 30.44 | 0% | 0.997 |
|  | 002_o_/110_t_ | 35.28 |  |  |

**Table S1. GIXRD peak deconvolution result – Aspect ratio**

| Composition | c-lattice parameter | a-lattice parameter | Aspect ratio | Unit cell volume |
| --- | --- | --- | --- | --- |
| Zr 50% | 5.056 | 5.129 | 1.014 | 133.02 Å^3^ |
| Zr 80% | 5.064 | 5.095 | 1.006 | 131.50 Å^3^ |
| Zr 100% | 5.087 | 5.072 | 0.997 | 130.85 Å^3^ |

**Table S2. GIXRD peak deconvolution result – unit cell volume**

**Note S1. Extraction of lattice parameters, aspect ratio, and unit-cell volume from GIXRD peak deconvolution.**

The structural parameters listed in Tables S1 and S2 were determined from the deconvoluted GIXRD peak positions shown in Figure S2. In particular, the ${111}_{o}/{101}_{t}$ and ${002}_{o}/{110}_{t}$ reflections were used to estimate the lattice parameters and the resulting aspect ratio of the HZO-based films.

The Bragg angles for the deconvoluted ${002}_{o}$ and ${111}_{o}$ peaks were obtained from the fitted $2\theta$ peak positions according to

|  | $\theta=\frac{2\theta}{2}$ | (S1) |
| --- | --- | --- |

The corresponding interplanar spacings were then calculated using Bragg’s law,

|  | $d_{hkl}=\frac{\lambda}{2\sin\theta}$ | (S2) |
| --- | --- | --- |

where $\lambda=1.5406$ Å for Cu K$\alpha$ radiation. Therefore,

|  | $d_{002}=\frac{1.5406}{2\sin\theta_{002}},d_{111}=\frac{1.5406}{2\sin\theta_{111}}$ | (S3) |
| --- | --- | --- |

To estimate the lattice parameters, the orthorhombic lattice relation was applied. For the ${002}_{o}$ reflection,

|  | $d_{002}=\frac{c}{2}$ | (S4) |
| --- | --- | --- |

which gives

|  | $c=2d_{002}$ | (S5) |
| --- | --- | --- |

For the ${111}_{o}$ reflection, the general orthorhombic relation is written as

|  | $\frac{1}{d_{111}^{2}}=\frac{1}{a^{2}}+\frac{1}{b^{2}}+\frac{1}{c^{2}}$ | (S6) |
| --- | --- | --- |

Assuming pseudo-symmetry in HZO, namely $a\approx b$, this expression can be simplified as

|  | $\frac{1}{d_{111}^{2}}=\frac{2}{a^{2}}+\frac{1}{c^{2}}$ | (S7) |
| --- | --- | --- |

from which the in-plane lattice parameter a was determined. Under the same pseudo-symmetry approximation, b was taken to be equal to a.

The aspect ratio was then calculated as

|  | $\text{Aspect ratio}=\frac{2c}{a+b}$ | (S8) |
| --- | --- | --- |

which reduces to $c/a$ when $a=b$. The unit-cell volume was calculated as

|  | $V=a\cdot b\cdot c$ | (S9) |
| --- | --- | --- |

using the extracted lattice parameters. Based on this procedure, the aspect ratio and unit-cell volume values reported in Tables S1 and S2 were obtained for the Hf_0.5_Zr_0.5_O_2_, Hf_0.2_Zr_0.8_O_2_, and pure ZrO_2_ films.


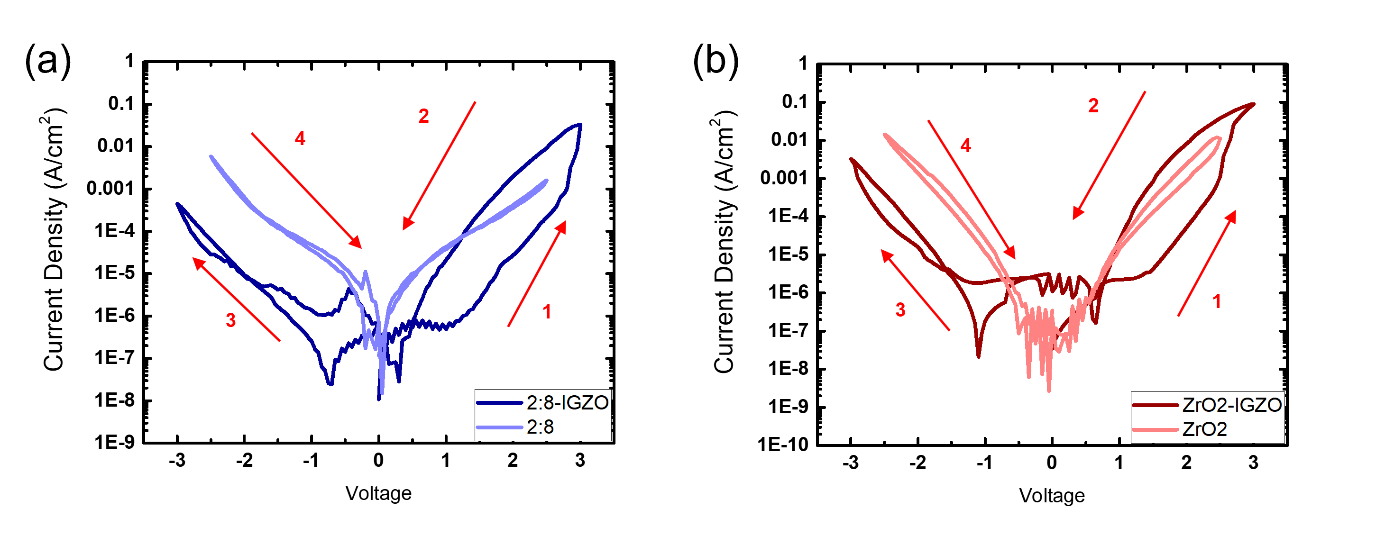


**Figure S3.** **Enhancement of memristive current hysteresis by inserting a 2 nm-thick a-IGZO interlayer on the antiferroelectric films.** Current density–voltage (J-V) hysteresis curves of bare antiferroelectric capacitors and the corresponding IGZO-integrated stacks for (a) Hf_0.2_Zr_0.8_O_2_ and (b) ZrO_2_. In each panel, the darker trace represents the AFTJ structure containing a $\sim$2 nm-thick a-IGZO interlayer, whereas the lighter trace corresponds to the capacitor without IGZO; the numbered arrows indicate the voltage sweep sequence. Insertion of the a-IGZO layer markedly enlarges the hysteretic current window and memory margin in both compositions while maintaining on-state current densities above ${10}^{-2}$ A/cm^2^. The maximum $I_{\mathrm{on}}/I_{\mathrm{off}}$ values reach 120 at 1.4 V for the Hf_0.2_Zr_0.8_O_2_-IGZO stack and 233 at 1.65 V for the ZrO_2_-IGZO stack, confirming that the ultrathin a-IGZO interlayer effectively enhances the memristive response of the antiferroelectric capacitor platform.


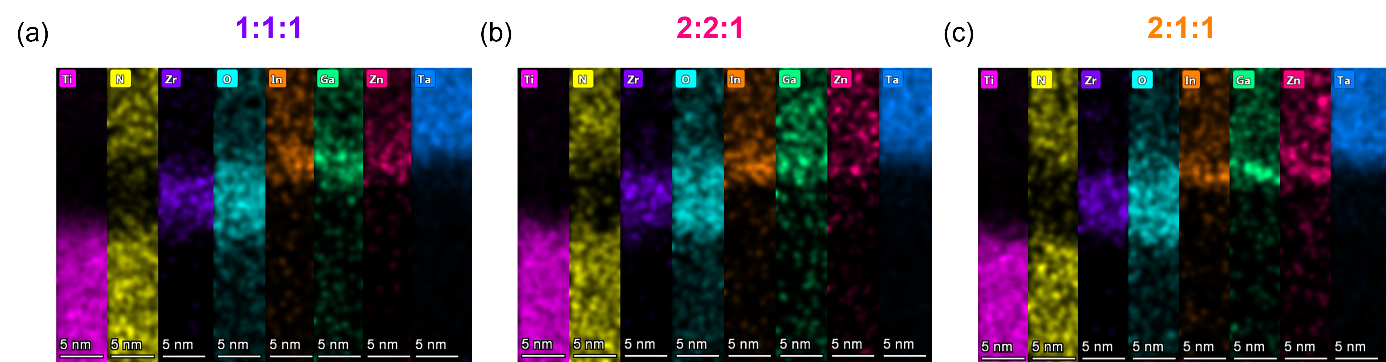


**Figure S4.** Cross-sectional EDS elemental mapping images of the engineered ZrO_2_ AFTJ stacks with a-IGZO layers of different stoichiometries: **(a)** 1:1:1, **(b)** 2:2:1, and **(c)** 2:1:1. In all cases, the Ti, N, Zr, O, In, Ga, Zn, and Ta signals resolve the vertically stacked TiN/ZrO_2_/IGZO/TaN structure. Scale bars, 5 nm.


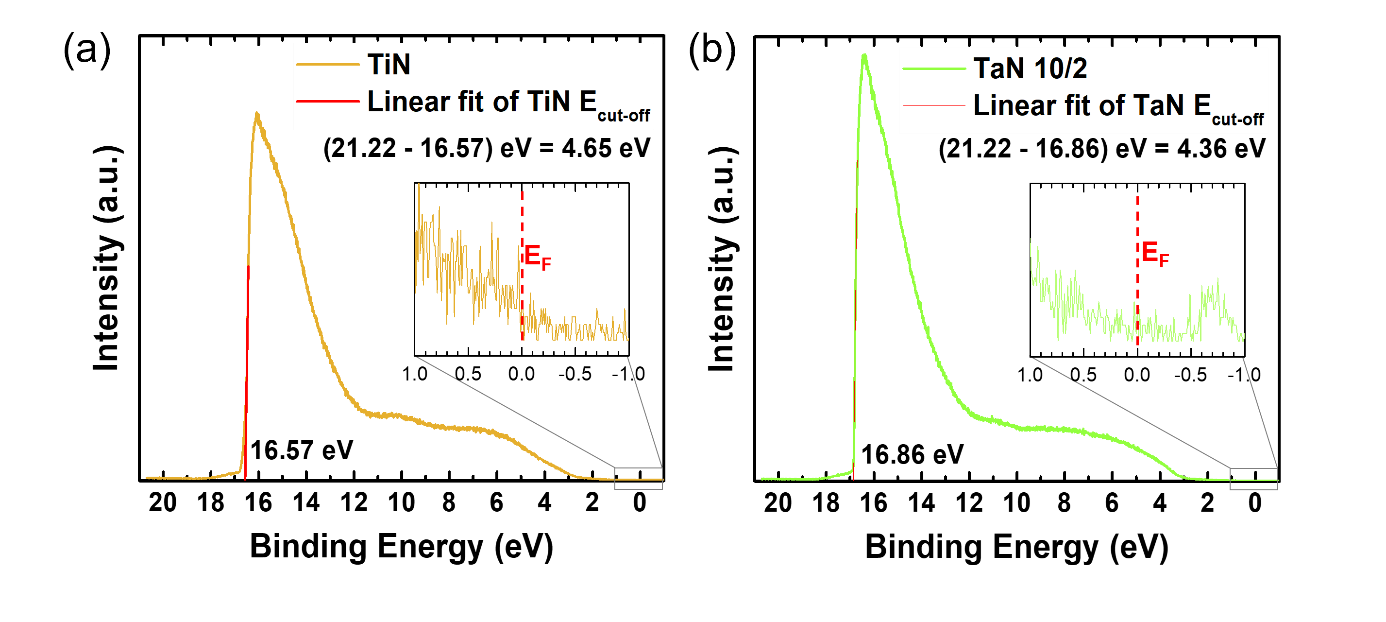


**Figure S5.** Ultraviolet photoelectron spectroscopy (UPS) spectra used to determine the work functions of the nitride electrodes: **(a)** TiN and **(b)** TaN. The work function ($\Phi$) was extracted from the secondary electron cutoff ($E_{\mathrm{cut}-\mathrm{off}}$) using $\Phi=h\nu-(E_{\mathrm{cut}-\mathrm{off}}-E_{F})$, where $h\nu=21.22$ eV for the He I source. The extracted work functions are 4.65 eV for TiN and 4.36 eV for TaN, indicating a work-function difference of 0.29 eV between the two electrodes.


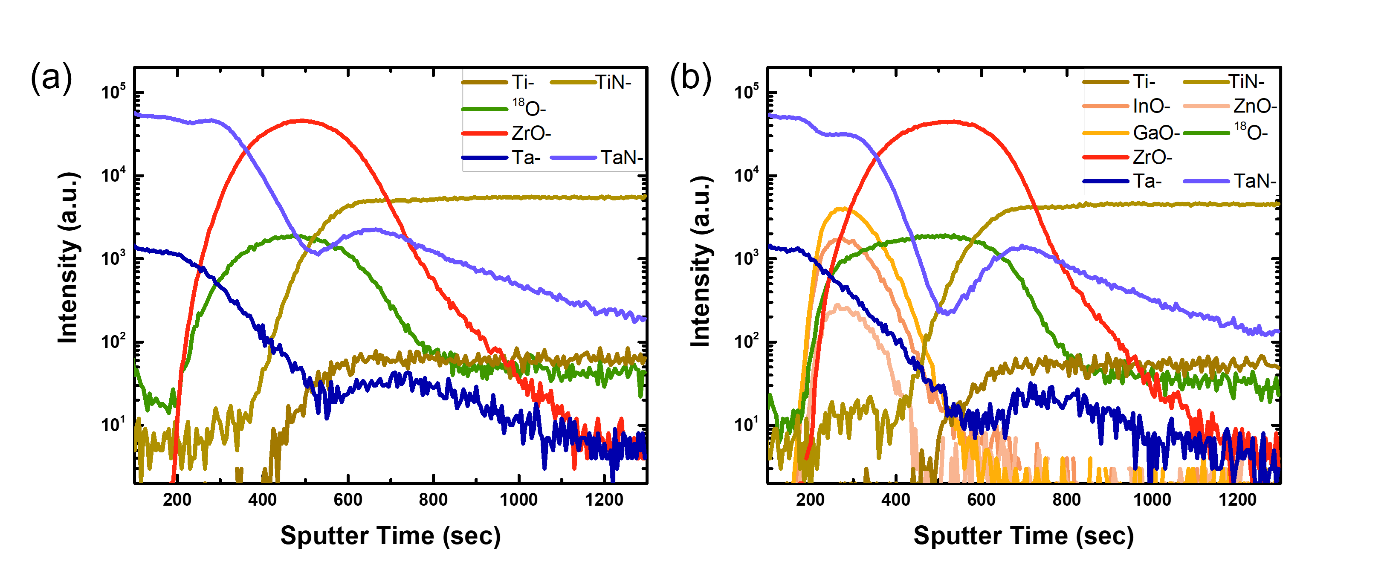


**Figure S6.** ToF-SIMS depth profiles comparing **(a)** the TiN/ZrO_2_/TaN capacitor and **(b)** the TiN/ZrO_2_/a-IGZO/TaN AFTJ stack. In the IGZO-integrated structure, additional InO^-^, GaO^-^, and ZnO^-^ signals appear between the TaN- and ZrO_x_-dominated regions, directly confirming the insertion of the ultrathin a-IGZO interlayer between the ZrO_2_ AFE film and the TaN top electrode. The overall evolution of the Ti-, Ta-, ZrO-, and oxygen-related signals is consistent with the intended vertical stack sequence and supports the structural integrity of the engineered AFTJ.


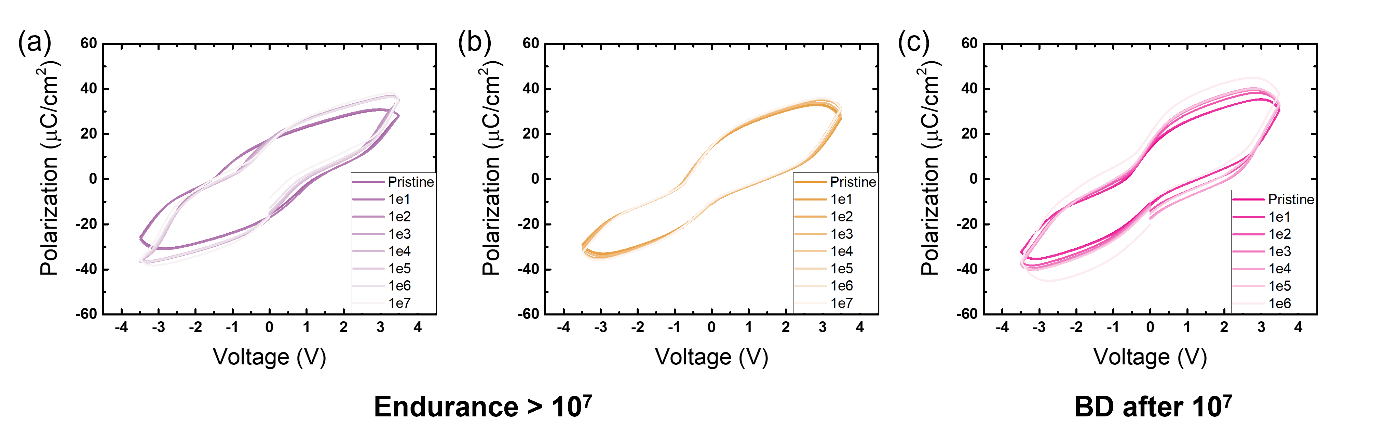


**Figure S7.** Evolution of polarization–voltage (\(P\)–\(V\)) hysteresis curves during fatigue cycling for ZrO_2_-based AFTJs with different IGZO compositions: **(a)** 1:1:1-ZrO_2_, **(b)** 2:2:1-ZrO_2_, and **(c)** 2:1:1-ZrO_2_. The P–V loops were recorded from the pristine state through repeated electric-field cycling up to ${10}^{7}$ cycles, revealing the wake-up and subsequent fatigue behavior of each device. The 1:1:1- and 2:2:1-ZrO_2_ AFTJs retain stable polarization characteristics beyond ${10}^{7}$ cycles, whereas the 2:1:1-ZrO_2_ device undergoes dielectric breakdown after ${10}^{7}$ cycles, indicating its reduced endurance under repeated operation.

**
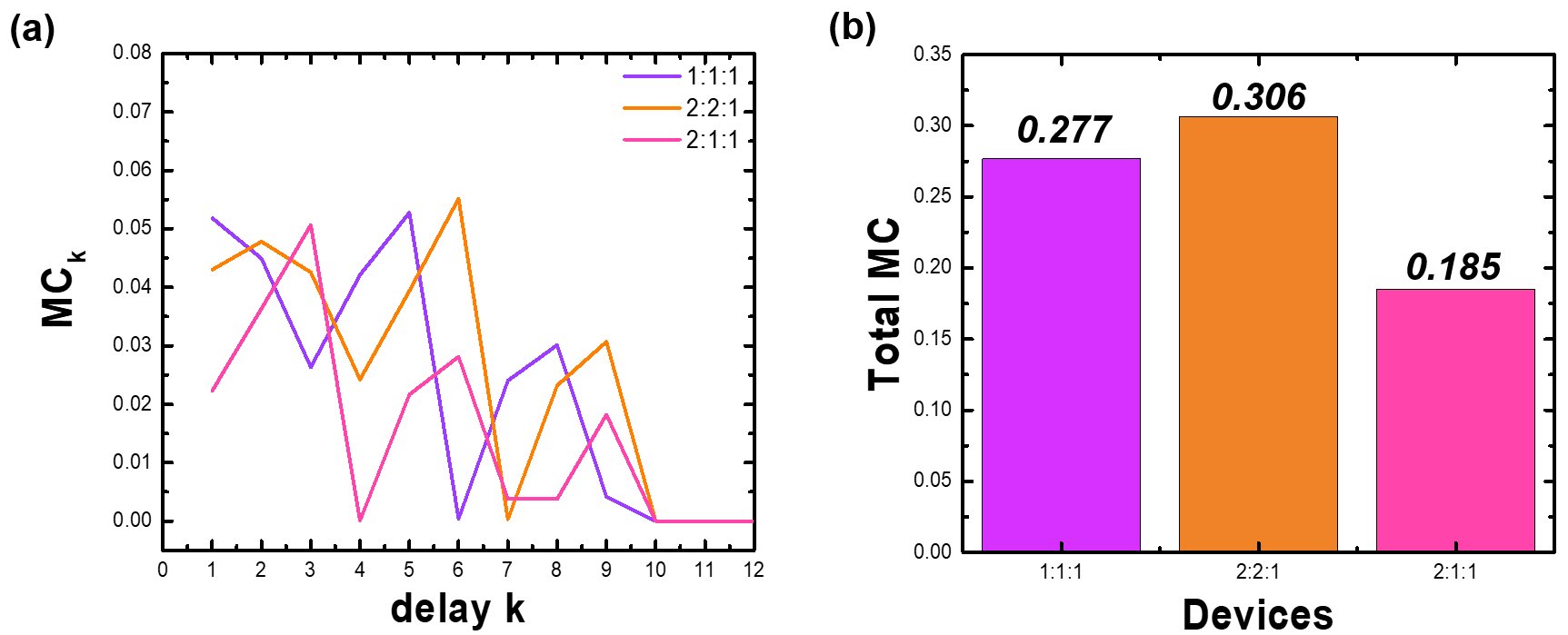
**

**Figure S8.** **Linear memory capacity analysis of ZrO_2_-based AFTJ reservoirs with different IGZO stoichiometries.** (a) Delay-dependent memory capacity MC_k_ as a function of delay k for the 1:1:1-ZrO_2_, 2:2:1-ZrO_2_, and 2:1:1-ZrO_2_ AFTJ reservoirs. MC_k_ was calculated as the squared correlation between the delayed random input u(n−k) and the reconstructed output obtained from the reservoir state X(n) using a trained linear readout. (b) Total memory capacity calculated by summing MC_k_ over the evaluated delay range.

Linear memory capacity analysis was performed to further quantify the sequence-level fading-memory capability of the ZrO_2_-based AFTJ reservoirs with different IGZO stoichiometries. In contrast to multibit-state distinguishability, memory capacity evaluates how effectively past input information can be reconstructed from the present reservoir state. An independent and identically distributed random input sequence u(n) was applied to the ngspice-based AFTJ reservoir model using the temporal masking framework. The resulting transient current responses were converted into reservoir state vectors X(n), and a linear readout was trained to reconstruct delayed inputs u(n−k). For each delay k, the delay-dependent memory capacity was calculated as MC_k_ = corr^2^[u(n−k), ŷ_k(n)_], where ŷ_k(n)_ is the reconstructed output. The total memory capacity was obtained by summing MC_k_ over the evaluated delay range [1,2]. The optimized 2:2:1-ZrO_2_ AFTJ exhibits the highest total memory capacity, indicating that past input information is more effectively preserved and reconstructed from its transient current states under the same reservoir protocol.


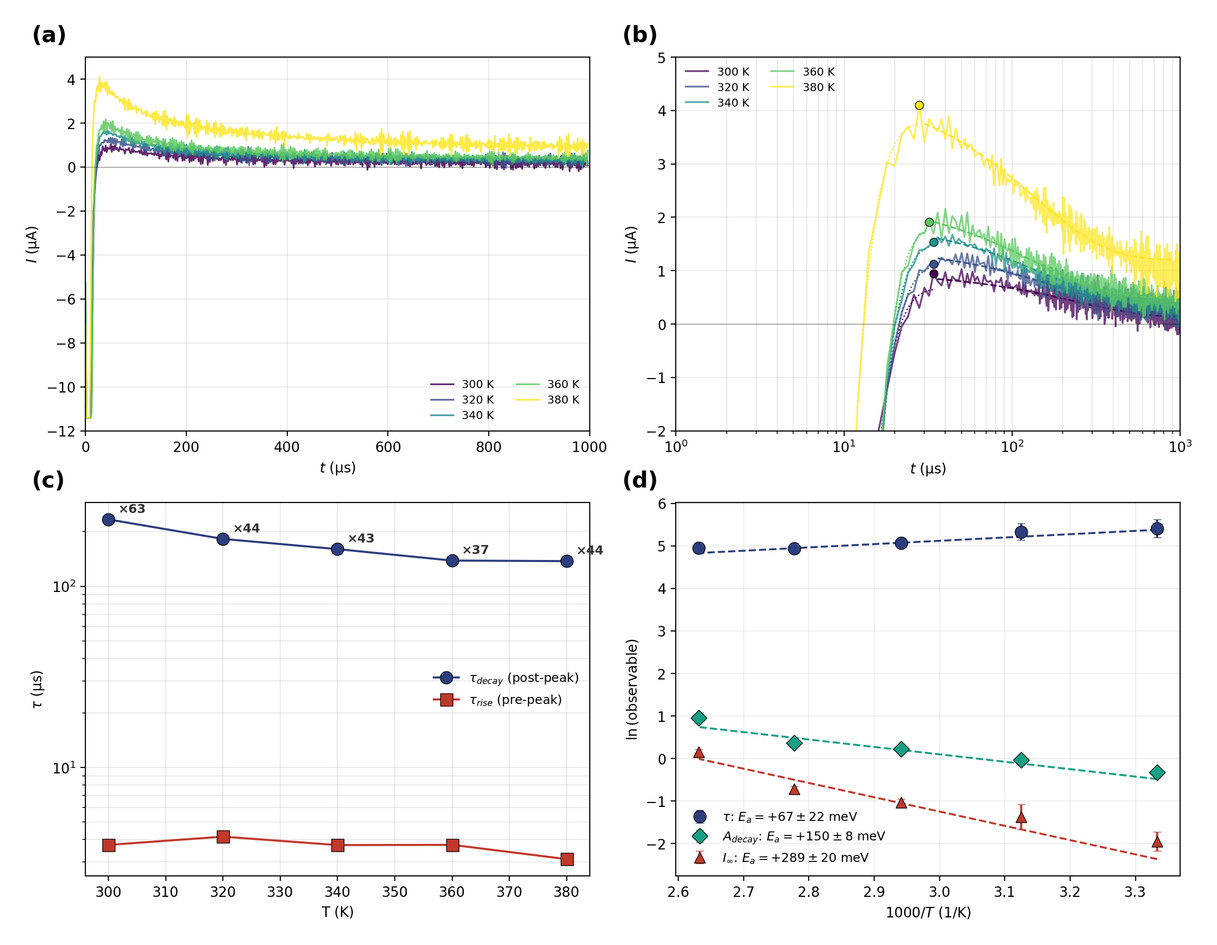


**Figure S9. Temperature-dependent post-peak relaxation of the optimized 2:2:1-ZrO_2_ AFTJ.** To separate the volatile AFE polarization-relaxation contribution from thermally activated transport, transient current responses were recorded at 300, 320, 340, 360, and 380 K under identical pulse conditions (3.5 V, 100 µs write; 1.8 V, 1 ms read). Five repetitions per temperature were ensemble-averaged, and the standard error of the mean (SEM) across these repetitions was retained as a measure of the inter-cycle noise at each time point. The time origin was defined as the read-pulse onset. The initial interval of the read window is saturated by the PMU current range (±10 µA) and corresponds to the rapid transient at the write→read voltage transition, which combines stack RC discharge and the fast polarization displacement current. The amplitude and time constant within this saturated interval are not accessible from the present measurement; the analysis is accordingly restricted to the resolvable segment following the positive zero-crossing. The resolvable trace is divided into a pre-peak rising segment (recovery from the current-saturation interval up to the transient current peak) and a post-peak decaying segment (fading relaxation); the full transient including the saturated interval is shown **in Figure S9a**, and the resolvable positive body with the fits is shown in **Figure S9b**. Each segment was fitted independently with a single-exponential model,

| *I*_rise_(*t*) = *I*_p_ − *A*_rise_ · exp(−*t* / τ_rise_) | (S10a) |
| --- | --- |
| *I*_decay_(*t*) = *I*_∞_ + *A*_decay_ · exp(−*t* / τ_decay_) | (S10b) |

where *I*_p_ denotes the post-write transient peak current and *I*_∞_ denotes the asymptotic current. No frequency-domain filtering was applied to the averaged traces. The fit minimized a weighted least-squares cost in which each time point was weighted by the inverse of its SEM-derived variance, such that less reproducible regions of the transient contributed less to the parameter estimates. Confidence intervals for the fit parameters were obtained from 200 nonparametric bootstrap resamples of the time-domain residuals. A single-exponential and a double-exponential model were compared using the Akaike information criterion (AIC); the average difference across temperatures, ΔAIC = +4, did not favor the double-exponential model within the present 1 ms read window. At this stage of the analysis, the extracted τ_decay_ represents an effective relaxation parameter that may convolve contributions from distributed polarization relaxation, interfacial charge redistribution, and trap-related dynamics.

In **Figure S9c**, τ_rise_ ≈ 3–4 µs shows no significant temperature dependence and reflects the recovery time from the current-saturation interval into the resolvable positive transient; it is not assigned to a thermally activated process. τ_decay_ = 138–234 µs lies more than 1.5 decades above τ_rise_ across the entire temperature range, indicating that capacitive discharge cannot account for the slow relaxation channel**.** **Figure S9d** summarizes the Arrhenius behavior of four independent observables. The post-peak time constant gives an activation energy of *E*_a_ (τ) = 67 ± 22 meV, lying within the range of kinetic barriers reported for field-induced antiferroelectric phase transitions in Zr-rich HZO [3,4].

The capacitive partition of the read pulse provides a physical constraint that narrows this interpretation. In the present stack, the 1.8 V read pulse partitions to ≈ 1.9 MV/cm across the ZrO_2_ layer, which lies above the back-switching field (E_FE→AFE_ ≈ 1.5 MV/cm) of 6 nm ZrO_2_ (Figure S3) but remains below the forward switching field (E_AFE→FE_ ≈ 3.4 MV/cm). Under this read condition, the field-induced o-phase polarization formed during the write pulse is not quasi-static and instead relaxes toward the antiferroelectric ground state under a reduced driving force relative to the bias-free condition. Field-induced switching in HZO/ZrO_2_ thin films has been characterized as a nucleation-controlled process whose characteristic time scale lengthens monotonically as the driving force decreases [5], and a back-relaxation time scale on the order of 100 µs under the reduced read-bias driving force is therefore consistent with the expected slowdown relative to forward switching at the full write field. Within this picture, the slowly relaxing AFE polarization modulates the effective tunnel barrier at the ZrO_2_/IGZO interface, and the IGZO trap-mediated channel registers this modulation in the read current. τdecay is accordingly interpreted as dominantly rate-limited by AFE polarization back-relaxation, with *E*_a_(τ) = 67 ± 22 meV assigned to the kinetic barrier of the field-induced antiferroelectric phase transition under the read-bias driving force.

In contrast, the decay amplitude and the asymptotic current exhibit stronger thermal activation, with *E*_a_(*A*_decay_) = 150 ± 8 meV and *E*ₐ(*I*_∞_) = 289 ± 21 meV. The latter falls within the activation-energy range reported for the dominant oxygen-vacancy trap of a-IGZO (~0.32 eV) [6], supporting the interpretation that the long-time leakage is governed by IGZO-side trap-mediated conduction. *E*_a_(A_decay_) = 150 ± 8 meV is correspondingly attributed to the thermal scaling of the trap-mediated read current under a time-varying polarization-induced barrier rather than to a single activation barrier.

Taken together, the post-peak relaxation of the optimized 2:2:1-ZrO_2_ AFTJ is consistent with a stack-coupled transient in which (i) the slow back-relaxation of the field-induced o-phase polarization in the ZrO_2_ layer modulates the effective tunnel barrier at the ZrO_2_/IGZO interface and dominantly sets the resolved time scale (*E*_a_(τ) = 67 ± 22 meV) and (ii) the trap-mediated channel in the IGZO interlayer reads out this barrier modulation as a time-varying current and governs the steady-state read-current magnitude (*E*_a_(*I*_∞_) = 289 ± 21 meV) together with the thermal scaling of the decay amplitude (*E*_a_(A_decay_) = 150 ± 8 meV). The AFE polarization state thereby modulates the amplitude and effective timescale of this trap-mediated conduction channel, and the resulting state-dependent, history-dependent response underlies the fading-memory behavior exploited for reservoir computing in this device. The IGZO interlayer assumes a dual role: it sets the read-current magnitude through its trap-mediated conductance and contributes to the long-time activation energy, while the resolved sub-millisecond relaxation timescale carries the imprint of the underlying AFE polarization dynamics.


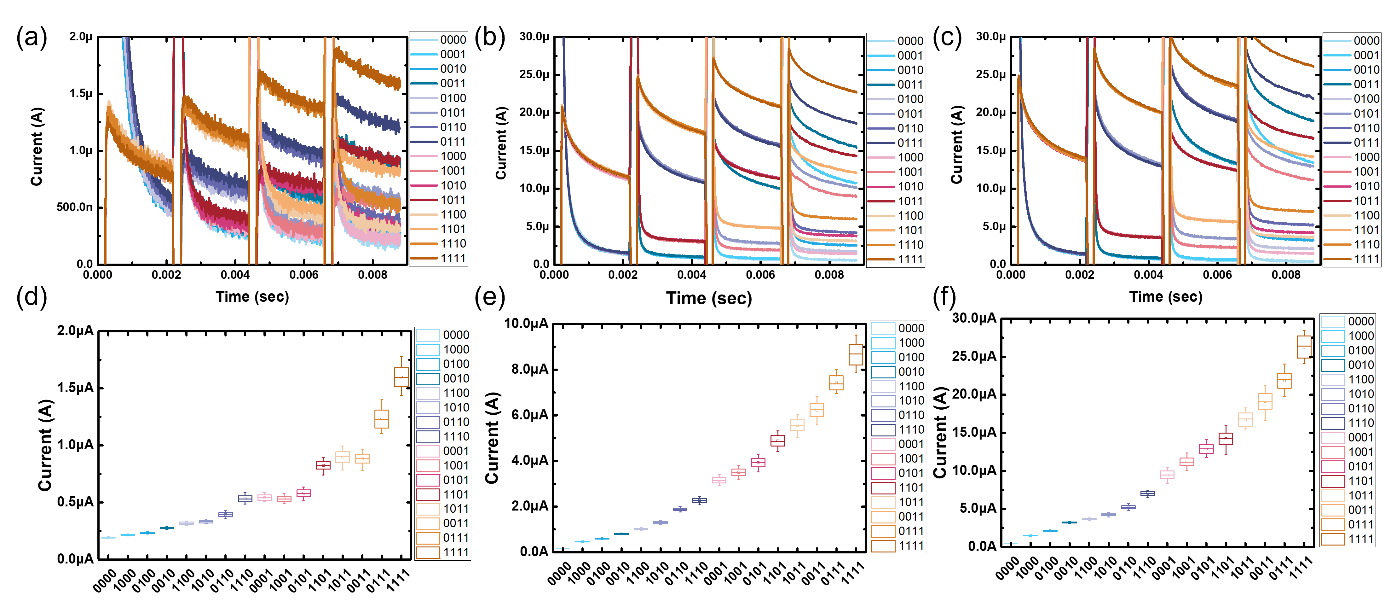


**Figure S10. 4-bit reservoir state current of AFTJ devices.**(a) 2:2:1-Hf_0.2_Zr_0.8_O_2_, (b) 1:1:1-ZrO_2_, and (c) 2:2:1-ZrO_2_ AFTJs showing transient current responses to 16 distinct 4-bit input patterns under the four-sequential read–write pulse scheme. (d–f) Corresponding current-state distributions extracted from 50 consecutive repetitions of the same 4-bit operation for the 2:2:1-Hf_0.2_Zr_0.8_O_2_, 1:1:1-ZrO_2_, and 2:2:1-ZrO_2_ devices, respectively. The ZrO_2_-based devices exhibit improved state separation compared with the Hf_0.2_Zr_0.8_O_2_-based device, and the 2:2:1-ZrO_2_ AFTJ shows the narrowest distribution spread and the clearest state discrimination.


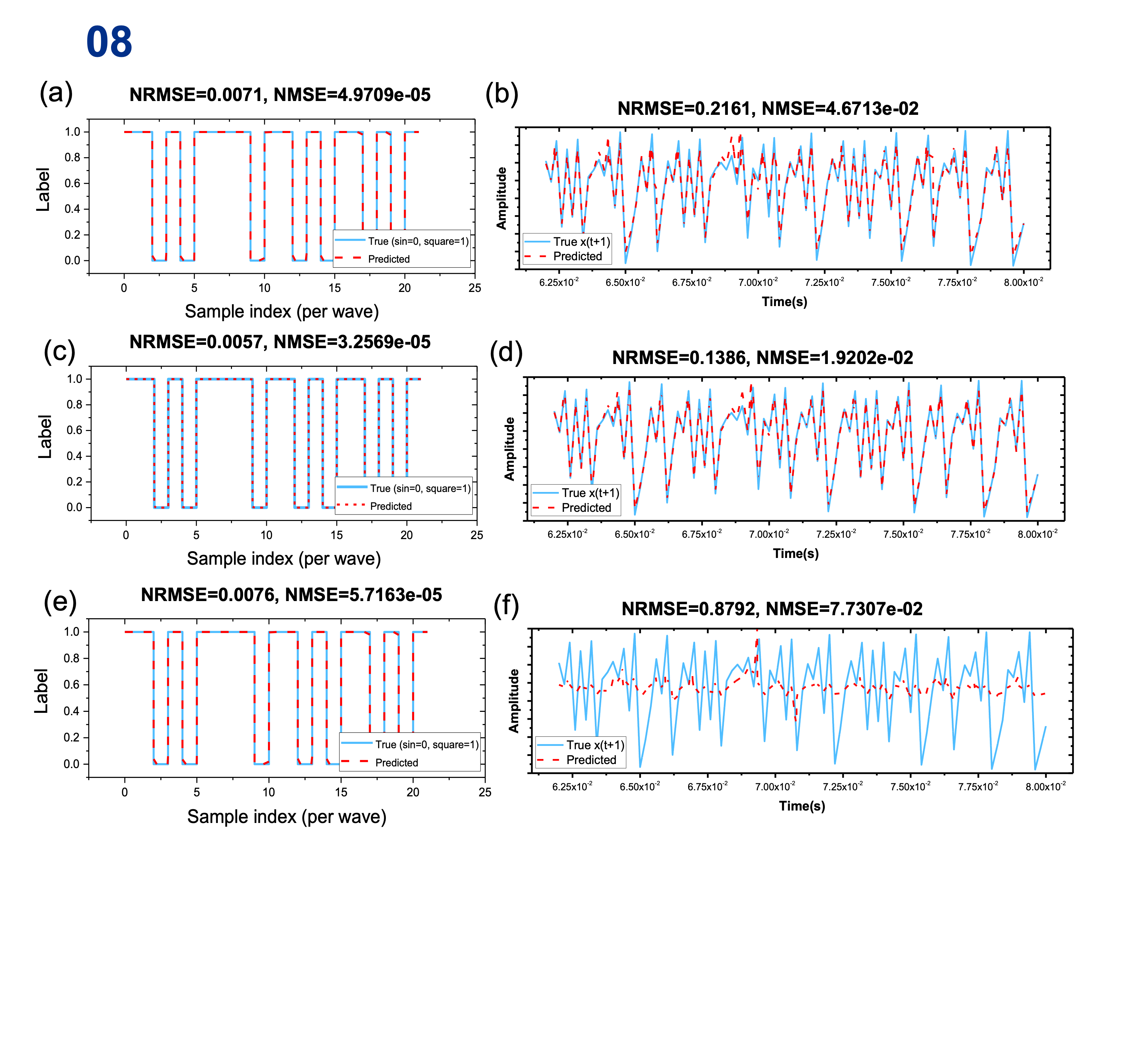


**Figure S11.** **Test results of the sinusoidal and square wave classification (left column) and one-step-ahead Hénon map prediction (right column) tasks utilizing the AFTJ physical reservoirs.** All results were obtained under an identical temporal masking configuration consisting of 9 virtual nodes and a mask length of 4. The panels display the true targets versus the predicted outputs for the 1:1:1-ZrO_2_ AFTJ (a, b), the optimal 2:2:1-ZrO_2_ AFTJ (c, d), and the 2:1:1-ZrO_2_ AFTJ (e, f), respectively. The excessively In-rich 2:1:1 composition fails to accurately trace the temporal dynamics due to its degraded state distinguishability and nonlinearity as discussed in the main text.


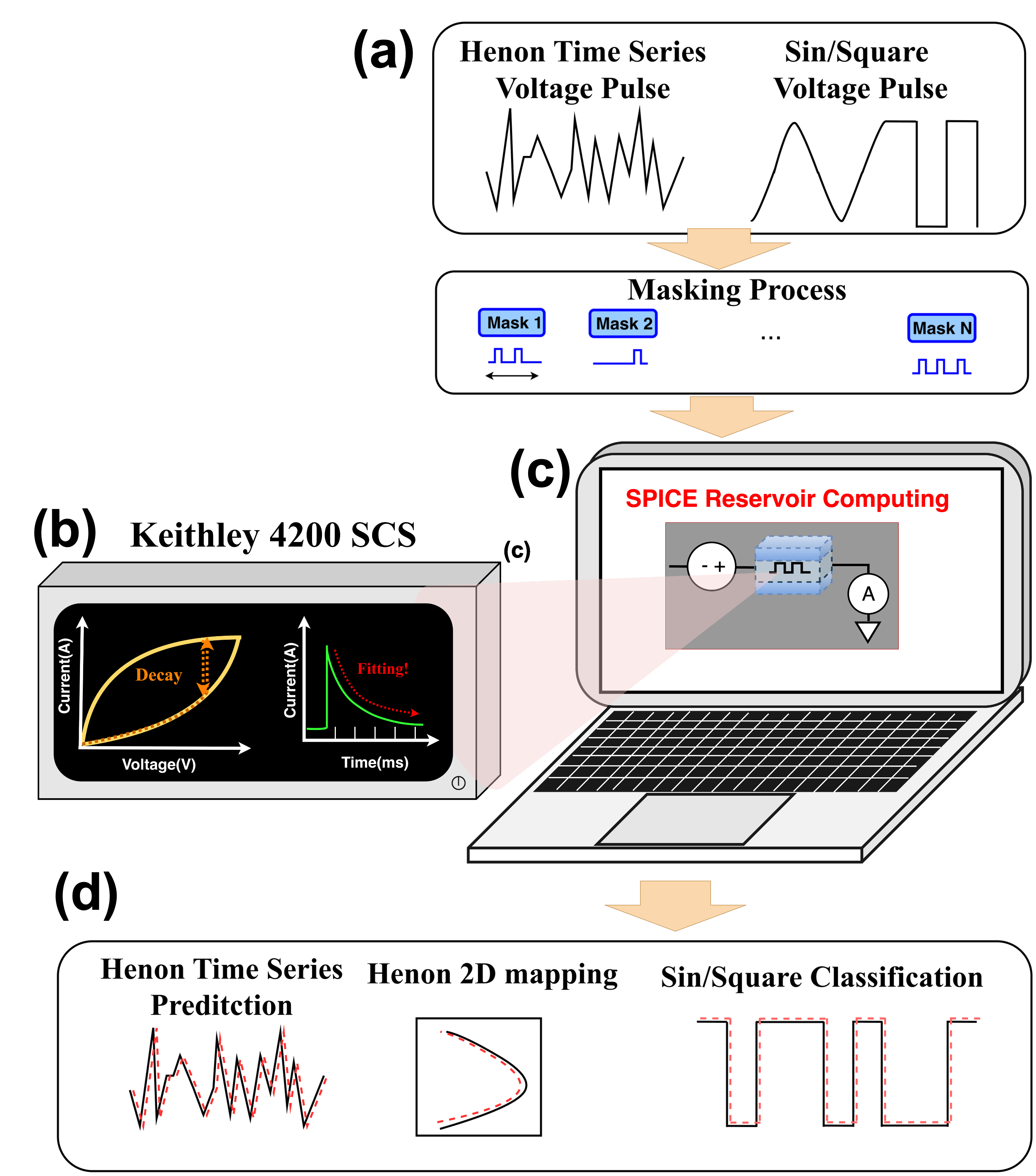


**Figure S12.** **Comprehensive schematic overview of the PRC simulation framework based on the AFTJ device.** (a) Masked pulse generation process where the original continuous signals such as sinusoidal waves, square waves, and the Hénon map time-series are multiplied by a random masking sequence to create time-multiplexed input voltage pulses. (b) Experimental device characterization setup utilizing a Keithley parameter analyzer to extract the essential electrical properties and temporal decay parameters of the fabricated AFTJ. (c) Reservoir simulation phase where the empirically extracted parameters are utilized to construct a virtual AFTJ model within the ngspice environment. The masked voltage pulses from the initial step are applied to this virtual device to dynamically measure the corresponding temporal current responses. (d) Schematic of the readout layer training and testing process showing how the collected current responses are utilized as reservoir states to optimize the trainable weights and ultimately evaluate the classification and prediction performance against the target signals.


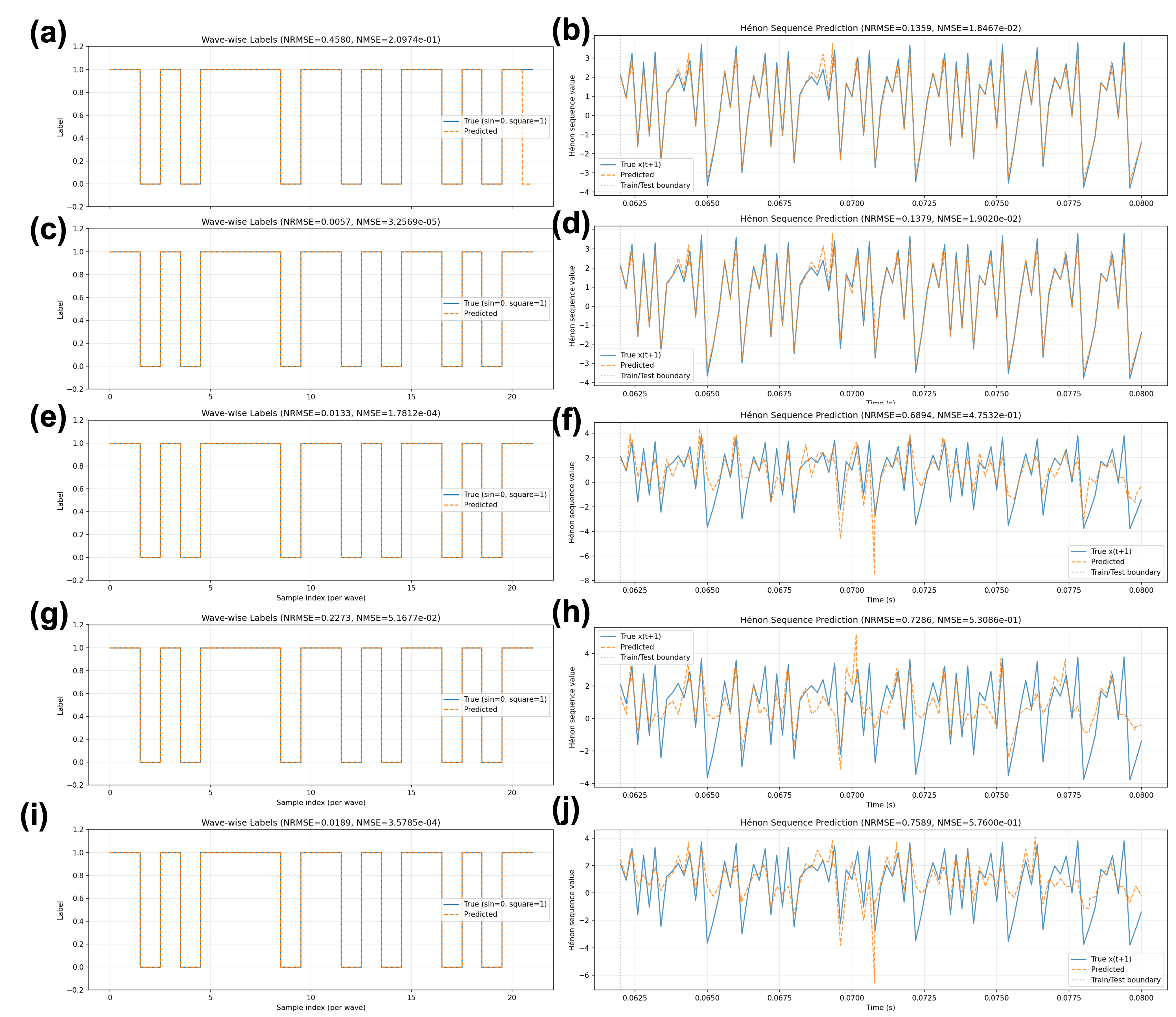


**Figure S13.** **Performance evaluation of the PRC system under various masking configurations.** The total reservoir dimension is fixed at 36 while the ratio of virtual nodes to mask length is systematically varied. The left column displays the classification results for the sinusoidal and square wave task and the right column shows the one-step-ahead forecasting results for the chaotic Hénon map. The configurations from top to bottom correspond to 12 virtual nodes with a mask length of 3 (a, b), 9 virtual nodes with a mask length of 4 (c, d), 6 virtual nodes with a mask length of 6 (e, f), 4 virtual nodes with a mask length of 9 (g, h), and 3 virtual nodes with a mask length of 12 (i, j). The normalized root mean square error and normalized mean square error values for each condition are explicitly evaluated within the respective panels to quantitatively compare the optimization of the temporal partitioning scheme. The optimal masking process identified through these simulations (9 virtual nodes with a mask length of 4 (c, d)) was further evaluated with an extended simulation duration of 0.4 s, as detailed in Figure 6 of the main text. Detailed numerical values for these performance metrics are summarized in Table S3.

| Masking Configuration | Task | Panel | NRMSE | NMSE |
| --- | --- | --- | --- | --- |
| 3 x 12 | Sinusoidal and Square | (a) | 0.458 | 2.10E-01 |
| 3 x 12 | Hénon Map | (b) | 0.1359 | 1.85E-02 |
| 4 x 9 | Sinusoidal and Square | (c) | 0.0057 | 3.26E-05 |
| 4 x 9 | Hénon Map | (d) | 0.1379 | 1.90E-02 |
| 6 x 6 | Sinusoidal and Square | (e) | 0.0133 | 1.78E-04 |
| 6 x 6 | Hénon Map | (f) | 0.6894 | 4.75E-01 |
| 9 x 4 | Sinusoidal and Square | (g) | 0.2273 | 5.17E-02 |
| 9 x 4 | Hénon Map | (h) | 0.7286 | 5.31E-01 |
| 12 x 3 | Sinusoidal and Square | (i) | 0.0189 | 3.58E-04 |
| 12 x 3 | Hénon Map | (j) | 0.7589 | 5.76E-01 |

**Table S3**. Quantitative summary of the prediction and classification performance under various spatial and temporal partitioning schemes. The normalized root mean square error and normalized mean square error are calculated for both the sinusoidal and square wave classification and the one-step-ahead Hénon map prediction tasks. The total dimension of the physical reservoir is maintained at 36 across all evaluated configurations to ensure a fair comparison. The optimal masking process identified from these simulations, utilizing 9 virtual nodes and a mask length of 4, was further evaluated using an extended simulation time of 0.4 s, as shown in Figure 6 of the main text. A comprehensive summary of the corresponding numerical values for these performance metrics is also provided.

| **Device Type** | **Stack** | **Device Area** | **Unit Operation Time** | **Energy per Unit Operation** | **Reference** |
| --- | --- | --- | --- | --- | --- |
| RRAM | Pd/TiOx/Ti | 25 μm^2^ | 100 ms | 108 nJ | [7] |
| RRAM | Ti/TiOx/TaOy/Pt | 0.75 μm^2^ | 3 ms | 6 nJ | [8] |
| RRAM | Pd/Au/WO_x_/W | 100 μm^2^ | 1.12 ms | 1.5 nJ | [9] |
| RRAM | W/WO_3_ /PEDOT:PSS/Pt | N/A | 1.5 ms | 9 nJ | [10] |
| FeFET | Pt/HZO/LSMO/LSAT | 800 μm^2^ | 110 s | 23.1 μJ | [11] |
| Ferroelectric Diode | Pt/BFO/SRO/STO | 2000 μm^2^ | 4 ms | 23.6 nJ | [12] |
| FTJ | TiN/HAO/SiO_2_/n+ Si | 100 μm^2^ | >130 ms | >22 nJ | [13] |
| FTJ | TiN/HAO/HfO_2_/n⁺ Si | 10000 μm^2^ | 1.01 ms | >800 nJ | [14] |
| Optoelectronics | ITO/ZnO/PbS QDs/  CuSCN/IDTBT/ZnO/Al | N/A | ~1 s | >420 nJ | [15] |
| Organic memristor | Al/Cl_2_CuPc/ITO/PET | 2500 μm^2^ | 5 s | >240 μJ | [16] |
| AFeFET | Mo/HZO/Mo/ZrO_2_/IGZO/ZrO_2_/Mo (double gate) | 400 μm^2^ | 3.1 ms | ~22.5 pJ | [17] |
| AFeFET | TiN/HZO/TiN/SiO_2_/p-Si | 100 μm^2^ | 20 ms | >800 nJ | [18] |
| Threshold Switch | Pt/Ag/ZrO_2_/TiN | 25 μm^2^ | 10.5 μs | 3.62 pJ | [19] |
| AFTJ | TaN/IGZO/ZrO_2_/TiN | 40000 μm^2^ | 2 μs | <480 pJ | This Work |
|  |  | 100 μm^2^ | ~192 ns | <~115 fJ | This Work  (projected) |

**Table S4**. Benchmark comparison of previously reported PRC devices for temporal data processing. Results devoted solely to classification tasks were excluded. For reports in which the energy per unit operation was not explicitly provided, the energy was calculated from the reported pulse width, voltage, and current values. Among the experimentally reported devices summarized here, the AFTJ reservoir device in this work shows the fastest unit operation time and the third-lowest energy consumption. If the scaling assumptions hold, the projected 100 μm^2^ AFTJ would reach <115 fJ per operation; however, this value should be regarded as an analytical projection rather than a directly benchmarked experimental result.

**Note S2. Measurement limitation, latency extrapolation, and energy estimation for the scaled AFTJ reservoir.** The energy consumption per unit operation of the AFTJ reservoir device was estimated from the pulse scheme used for temporal data processing tasks, including wave classification and Hénon map prediction. In the write step, analog real-valued inputs were encoded using voltage pulses with amplitudes ranging from 2.6 to 3.5 V and a pulse width of 0.18 μs. In the subsequent read step, a 1.8 V pulse with a duration of 1.8 μs was used.

The transient current measurements were performed using a Keithley 4200-SCS parameter analyzer equipped with 4225-PMU ultrafast pulse-measure units and 4225-RPM remote amplifier/switch modules. The timing specifications of the 4225-RPM impose a practical trade-off between measurable current range and temporal resolution. According to the Keithley technical data for the 10 V range, the 100 µA current range supports a recommended minimum pulse width of 920 ns, a minimum measurement window of 130 ns, and a settling time of 750 ns, whereas the 100 nA range requires a recommended minimum pulse width of 111 µs, a measurement window of 10 µs, and a settling time of 100 µs. Thus, the experimentally demonstrated 40,000 µm^2^ AFTJ, with a read current of approximately 25 µA, could be reliably characterized using the 1.8 µs read pulse. In contrast, under the same current-density scaling assumption, the read current of a projected 100 µm^2^ AFTJ would decrease to approximately 62.5 nA, placing the signal near the 100 nA range. Because the timing requirements in this low-current range are much longer than the projected sub-µs operation window, direct extraction of the decay constant for the projected 100 µm^2^ operating condition is not reliable in the present pulsed-measurement configuration. Therefore, the latency and energy values of the 100 µm^2^ AFTJ were estimated from the experimentally measured area-dependent scaling trend rather than from direct time-series operation under the projected operating condition.

Because the transient current response in both steps is nonlinear, the most rigorous evaluation of the consumed energy would require direct integration of the time-dependent current. However, owing to the limited number of data points that could be acquired within such a short time window using the pulsed-measurement setup, accurate time integration was not feasible in the present work. Therefore, to avoid overestimating the performance and to provide a conservative estimate of the energy consumption, we assumed a rectangular current response in which the peak current measured in each pulse segment was maintained throughout the corresponding pulse duration. For this reason, the energy values in the main text and Table S4 are presented using inequality signs.

Using the average write voltage of 3.0 V, the peak current measured during the 0.18 μs write pulse was 739 μA. Likewise, the peak current measured during the 1.8 V, 1.8 μs read pulse was 25 μA. Based on the rectangular-response approximation, the write energy is estimated as $E_{\mathrm{write}}=V_{\mathrm{write}}I_{\mathrm{write},\mathrm{peak}}t_{\mathrm{write}}=3.0\times739\text{ }\mu A\times0.18\text{ }\mu s=3.9906\times{10}^{-10}\text{ }J$, corresponding to 399.06 pJ. The read energy is similarly estimated as $E_{\mathrm{read}}=V_{\mathrm{read}}I_{\mathrm{read},\mathrm{peak}}t_{\mathrm{read}}=1.8\times25\text{ }\mu A\times1.8\text{ }\mu s=8.1\times{10}^{-11}\text{ }J$, corresponding to 81 pJ. Accordingly, the total average energy consumption per unit operation is conservatively estimated as $E_{\mathrm{unit}}=E_{\mathrm{write}}+E_{\mathrm{read}}=4.8006\times{10}^{-10}\text{ }J$, or 480.06 pJ.

To further estimate the energy scalability of the AFTJ reservoir, actual devices with areas ranging from 40,000 to 400 μm^2^ were fabricated using a photolithography patterning process, and their decay time constants were experimentally extracted. Among the fitted parameters, $\tau_{1}$, which showed the clearest area dependence, was selected for scaling analysis. Specifically, the four experimentally obtained $\left( A , \tau_{1} \right)$ data points were fitted on a $\log_{10}A$-$\log_{10}\tau_{1}$ plot using a linear relation, $\log_{10}\tau_{1}=m\log_{10}A+b$, which corresponds to the power-law form $\tau_{1}={10}^{b}A^{m}$. Using the fitted exponent $m=0.3909565$ and taking the measured 40,000 μm^2^ device with $\tau_{1}=2.0\times{10}^{-6}$ s as the reference point, the time constant of a 100 μm^2^ device was extrapolated according to $\tau_{1}(100)=2.0\times{10}^{-6}\times(100/40000)^{0.3909565}\approx1.92\times{10}^{-7}$ s. Based on this fitting, the unit operation time of a scaled 10 × 10 μm^2^ (100 μm^2^) device was therefore projected to be approximately 192 ns.

Using this projected operation time and the same conservative energy-estimation procedure described above, the energy consumption of the scaled device was estimated to be 115 fJ per operation. For fairness in the benchmark comparison, this projected value for the hypothetically scaled device was not included in the benchmark plot in Figure 7d, and only experimentally measured devices were used for the direct comparison.

**Note S3.**

*MNIST Image Classification*: MNIST images were preprocessed to a resolution of 22 × 20 pixels. Subsequently, each 20-pixel row was divided into five 4-pixel segments to form 16 distinct 4-bit input patterns. Each 4-bit pattern was then mapped one-to-one onto one of the 16 experimentally distinguished state current values of the AFTJ. For each of the 22 rows, five time-multiplexed reservoir sampling points were collected yielding a 22 × 5 reservoir state matrix for a single image. Flattening this matrix into a one-dimensional structure yields the 110-dimensional reservoir state vector. This signifies that the two-dimensional spatial features of the original image were transformed into states within the temporal reservoir space through the physical dynamics of the AFTJ device.

*Temporal Task Configuration and Simulation Protocol*: Both the Hénon map prediction and the sinusoidal and square wave classification tasks were conducted using an identical temporal protocol. All simulations were performed in the ngspice environment. The total simulation duration was set to 0.08 s and discretized into 40,000 time steps. The initial interval from 0 s to 0.008 s was used as a washout period to eliminate the influence of initial reservoir conditions. The subsequent interval from 0.008 s to 0.062 s was used as the training phase for learning the readout weights. The final interval from 0.062 s to 0.08 s was reserved as the test phase for performance evaluation. This temporal partitioning was consistently applied to both the Hénon map prediction and the sinusoidal and square wave classification tasks. For the masking combination (mask length:4, number of masks: 9) which exhibited the lowest error rate, the total sequence length was extended by a factor of five to 0.4 s to verify the prediction stability. In this extended simulation, the temporal ratios for the washout, train, and test phases were adjusted to 5%, 25%, and 70% respectively.

*Hénon Map Definition and Prediction Scheme*: The Hénon map is a representative discrete-time nonlinear dynamical system that exhibits chaotic behavior because of its extreme sensitivity to initial conditions, despite being governed by simple deterministic equations. In this study, the state evolution of this system is defined by the following two coupled equations^[20]^:

|  | $x\left( k+1 \right)=1-ax^{2}\left( k \right)+y\left( k \right)$ | (S11) |
| --- | --- | --- |
|  | $y\left( k+1 \right)=b x(k)$ | (S12) |

Here, a and b are the core parameters determining the system behavior; they were set to 1.4 and 0.3 respectively to induce typical and stable chaotic dynamics. As revealed in the equations above determining the state at a specific time point $x\left( k+1 \right)$ simultaneously requires not only the nonlinear squared term of the immediately preceding state $x\left( k \right)$ but also the information of $y\left( k \right)$ which inherently contains the state from one step further in the past $x\left( k-1 \right)$. Precisely due to this mathematical characteristic the Hénon map functions as a rigorous benchmark to verify whether the target system possesses the STM characteristic to maintain past temporal information without loss and the nonlinearity to map input data into high-dimensional space. In this study, the generated one-dimensional Hénon time-series $x\left( k \right)$ was converted into continuous voltage pulses according to the previously established temporal protocol and applied as an input signal to the virtual AFTJ reservoir. For the prediction model a one-step-ahead prediction scheme was applied where the readout layer is trained to estimate the value of $x\left( k+1 \right)$ at the very next time point based on the time-dependent current response of the AFTJ extracted at the current time point $k$ namely, the reservoir state. This setup evaluates whether the fading memory effect naturally derived from the physical relaxation process of the AFTJ device itself without artificial external control can simulate and predict the temporal dynamics of a complex chaotic system.

*Sinusoidal and Square Wave Classification*: For waveform classification, sinusoidal and square wave signals were used as input patterns. In accordance with the previously established temporal protocol the total simulation duration of 0.08 s was discretized into 40,000 time steps consequently allocating a length of 2 μs to each single time step. Both waveforms were randomly generated and arranged in continuous sequences with an identical period of 8 time steps, corresponding to 16 μs. This randomly generated continuous pulse sequence underwent a specific masking process before being applied as an input signal to the virtual AFTJ reservoir.

Sinusoidal inputs induced smoothly varying current responses whereas square wave inputs caused abrupt current transitions due to sudden voltage fluctuations. It is crucial to note that these morphological differences in the input signals were amplified through the intrinsic nonlinear response and relaxation dynamics of the AFTJ device, forming clearly distinguishable reservoir state distributions. Ultimately, the readout layer was trained to accurately discriminate and classify the target shape of the original voltage pulse as either sinusoidal or square based solely on the magnitude of the derived temporal current responses without requiring complex computations.

*Temporal Task Configuration:* To assess the practical edge-computing potential of the PRC system real-world financial time-series forecasting was performed using the PHLX SOX index. This evaluation was conducted based on weekly index data collected from 1994 to February 2026. The complex and unstructured actual index values were linearly mapped to voltage pulse amplitudes ranging from 1.8 V to 3.8 V to match the operating region of the optimized 2:2:1-ZrO_2_ AFTJ device.

To extract the temporal features inherent in the data the previously established masking process was applied to extract temporal features through the device and specifically a configuration with 9 virtual nodes and a mask length of 4 was applied. The entire transformed sequential data were divided into three distinct phases and processed. The initial period from 1994 to November 1995 was allocated as a washout phase to stabilize the initial reservoir states of the system and was discarded. The subsequent section from December 1995 to July 2015 was used as a training phase to learn the readout weights and finally the data from August 2015 to February 2026 was assigned as a testing phase to verify the final prediction performance. The readout layer was designed to predict future index trends based solely on the temporal current responses derived from the AFTJ device without complex computations and the final prediction accuracy of the system was quantitatively calculated through normalized mean square error measurements.

*Reservoir Dimension Configuration*: In PRC, a masking process is typically required to overcome the limited dimensionality of a single device and project input data into a high-dimensional space. Both waveforms were randomly generated and arranged in continuous sequences with an identical period of 8 time steps, corresponding to 16 μs. Specifically the original voltage pulse is combined with $N$ masks having a specific length $M$ and converted into a continuous masked input stream finely divided along the time axis. When this converted signal is applied to the virtual AFTJ device a unique temporal current response is derived for each virtual node section due to the intrinsic nonlinear response and fading memory characteristics of the device. The system sequentially samples these response current values to collect them as reservoir states and ultimately the readout layer performs training and testing processes to derive optimal weights based on the collected states targeting the original voltage pulse or other objectives.

Here $N$ represents the total number of virtual nodes spatially allocated for a single input signal while $M$ denotes the number of consecutive reservoir sampling steps extracted within each virtual node namely the masking length. For both the Hénon map prediction and waveform classification tasks the total reservoir dimension of the system which is the product of $N$ and $M$ was fixed at 36. Instead, to analyze the interaction between the spatial nonlinearity and the temporal fading memory effect provided by the AFTJ device the combination of $N$ and $M$ was systematically varied into nine configurations: (1, 36), (2, 18), (3, 12), (4, 9), (6, 6), (9, 4), (12, 3), (18, 2), and (36, 1). Identical input data and temporal protocols were consistently applied across all configuration groups to enable a fair comparison of how these changes in the partitioning ratio influence the computational performance of the system.

**References**

1. J. Dambre, D. Verstraeten, B. Schrauwen, S. Massar, “Information Processing Capacity of Dynamical Systems,” *Scientific Reports* 2 (2012): 514.
<https://doi.org/10.1038/srep00514>

2. G. Tanaka, T. Yamane, J. B. Héroux, et al., “Recent Advances in Physical Reservoir Computing: A Review,” *Neural Networks* 115 (2019): 100-123.
<https://doi.org/10.1016/j.neunet.2019.03.005>

3. M. H. Park, Y. H. Lee, T. Mikolajick, U. Schroeder, C. S. Hwang, “Review and Perspective on Ferroelectric HfO_2_-Based Thin Films for Memory Applications,” *MRS Communications* 8, no. 3 (2018): 795-808.

<https://doi.org/10.1557/mrc.2018.175>

4. M. Pešić, F. P. G. Fengler, L. Larcher, et al., “Physical Mechanisms behind the Field-Cycling Behavior of HfO_2_-Based Ferroelectric Capacitors,” *Advanced Functional Materials* 26, no. 25 (2016): 4601–4612.

<https://doi.org/10.1002/adfm.201600590>

5. A. Chouprik, E. Kondratyuk, “Polarization Switching Kinetics in Thin Ferroelectric HZO Films,” *Nanomaterials* 12, no. 23 (2022): 4126.
<https://doi.org/10.3390/nano12234126>

6. M. J. Mattsson, J. Lee, C. E. Malmberg, et al., “Shallow Trap States Control Electrical Performance of Amorphous Oxide Semiconductor Thin-Film Transistors,” *arXiv preprint* arXiv: 2602.06329 (2026).
<https://doi.org/10.48550/arXiv.2602.06329>

7. Y. Zhong, J. Tang, X. Li, et al., “A memristor-based analogue reservoir computing system for real-time and power-efficient signal processing,” *Nature Electronics* 5 (2022): 672-681.
<https://doi.org/10.1038/s41928-022-00838-3>

8. C. Du, F. Cai, M. A. Zidan, et al., “Reservoir Computing Using Dynamic Memristors for Temporal Information Processing,” *Nature Communications* 8, no. 1 (2017): 2204.
<https://doi.org/10.1038/s41467-017-02337-y>

9. Y. Zhong, J. Tang, X. Li, et al., “Dynamic Memristor-Based Reservoir Computing for High-Efficiency Temporal Signal Processing,” *Nature Communications* 12, no. 1 (2021): 408.
<https://doi.org/10.1038/s41467-020-20692-1>

10. T. Wang, H.-M. Huang, X.-X. Wang, X. Guo, “An Artificial Olfactory Inference System Based on Memristive Devices,” *InfoMat* 3, no. 7 (2021): 804-813.
<https://doi.org/10.1002/inf2.12196>

11. Z. Liu, Q. Zhang, D. Xie, et al., “Interface-Type Tunable Oxygen Ion Dynamics for Physical Reservoir Computing,” *Nature Communications* 14, no. 1 (2023): 7176.
<https://doi.org/10.1038/s41467-023-42993-x>

12. Z. Chen, W. Li, Z. Fan, et al., “All-Ferroelectric Implementation of Reservoir Computing,” *Nature Communications* 14, no. 1 (2023): 3585.
<https://doi.org/10.1038/s41467-023-39371-y>

13. Y. Park, E. Lim, S. Lee, et al., “Ferroelectric Memristor Crossbar Arrays for Highly Integrated Neuromorphic Computing System,” *Nano Energy* 141 (2025): 111137.
<https://doi.org/10.1016/j.nanoen.2025.111137>

14. J. K. Lee, Y. Park, E. Seo, et al., “Integrated Design of Electrically Configurable Ferroelectric and Redox-Based Memristors for Hardware-Implemented Reservoir Computing,” *Advanced Science* 12, no. 33 (2025): e05688.
<https://doi.org/10.1002/advs.202505688>

15. Z. Chen, P. Liang, T. Lin, et al., “Brain-Inspired Multi-Time Scale Reservoir Computing with Organic Optoelectronic Memristors,” *American Chemical Society Photonics* 13, no. 9 (2026): 2645–2652.
<https://doi.org/10.1021/acsphotonics.6c00162>

16. J. Zhou, W. Li, H. Zhu, et al., “Flexible Organic Memristor for Robust Physical Reservoir Computing in Diversified Dynamic Tasks,” *Advanced Functional Materials* 36, no. 31 (2026): e31718.
<https://doi.org/10.1002/adfm.202531718>

17. J. Kim, E. C. Park, W. Shin, et al., “Analog Reservoir Computing via Ferroelectric Mixed Phase Boundary Transistors,” *Nature Communications* 15, no. 1 (2024): 9147.
<https://doi.org/10.1038/s41467-024-53321-2>

18. T. Jung, D. Kim, G. Kim, et al., “Vertically Integrated In-Sensor Processing System Based on Three-Dimensional Reservoir for Artificial Tactile System,” *Energy & Environmental Materials* 8, no. 6 (2025): e70063.
<https://doi.org/10.1002/eem2.70063>

19. D. K. Lee, G. Noh, S. Oh, et al., “Crystallinity-Controlled Volatility Tuning of ZrO_2_ Memristor for Physical Reservoir Computing,” *InfoMat* 7, no. 2 (2025): e12635.
<https://doi.org/10.1002/inf2.12635>

20. M. Hénon, “A Two-Dimensional Mapping with a Strange Attractor,” *Communications in Mathematical Physics* 50, no. 1 (1976): 69-77.
<https://doi.org/10.1007/BF01608556>
